# Genetic analysis of Mendelian mutations in a large UK population-based Parkinson’s disease study

**DOI:** 10.1093/brain/awz191

**Published:** 2019-07-19

**Authors:** Manuela M X Tan, Naveed Malek, Michael A Lawton, Leon Hubbard, Alan M Pittman, Theresita Joseph, Jason Hehir, Diane M A Swallow, Katherine A Grosset, Sarah L Marrinan, Nin Bajaj, Roger A Barker, David J Burn, Catherine Bresner, Thomas Foltynie, John Hardy, Nicholas Wood, Yoav Ben-Shlomo, Donald G Grosset, Nigel M Williams, Huw R Morris

**Affiliations:** 1 Department of Clinical and Movement Neurosciences, UCL Queen Square Institute of Neurology, London, UK; 2 UCL Movement Disorders Centre, University College London, London, UK; 3 Department of Neurology, Institute of Neurological Sciences, Queen Elizabeth University Hospital, Glasgow, UK; 4 Population Health Sciences, University of Bristol, UK; 5 Institute of Psychological Medicine and Clinical Neurosciences, MRC Centre for Neuropsychiatric Genetics and Genomics, Cardiff University, Cardiff, UK; 6 University College London Hospitals NHS Foundation Trust, UK; 7 Institute of Neuroscience, University of Newcastle, Newcastle upon Tyne, UK; 8 Department of Clinical Neurosciences, University of Nottingham, UK; 9 Wellcome - MRC Cambridge Stem Cell Institute, University of Cambridge, Cambridge UK; 10 Department of Clinical Neurosciences, John van Geest Centre for Brain Repair, Cambridge, UK; 11 Reta Lila Weston Laboratories, Department of Molecular Neuroscience, UCL Institute of Neurology, London, UK

**Keywords:** Parkinson’s disease, genetics, phenotype, heterogeneity, prevalence

## Abstract

Our objective was to define the prevalence and clinical features of genetic Parkinson’s disease in a large UK population-based cohort, the largest multicentre prospective clinico-genetic incident study in the world. We collected demographic data, Movement Disorder Society Unified Parkinson’s Disease Rating Scale scores, and Montreal Cognitive Assessment scores. We analysed mutations in *PRKN *(parkin), *PINK1*, *LRRK2 *and *SNCA* in relation to age at symptom onset, family history and clinical features. Of the 2262 participants recruited to the Tracking Parkinson’s study, 424 had young-onset Parkinson’s disease (age at onset ≤ 50) and 1799 had late onset Parkinson’s disease. A range of methods were used to genotype 2005 patients: 302 young-onset patients were fully genotyped with multiplex ligation-dependent probe amplification and either Sanger and/or exome sequencing; and 1701 late-onset patients were genotyped with the *LRRK2* ‘Kompetitive’ allele-specific polymerase chain reaction assay and/or exome sequencing (two patients had missing age at onset). We identified 29 (1.4%) patients carrying pathogenic mutations. Eighteen patients carried the G2019S or R1441C mutations in *LRRK2*, and one patient carried a heterozygous duplication in *SNCA.* In *PRKN*, we identified patients carrying deletions of exons 1, 4 and 5, and P113Xfs, R275W, G430D and R33X. In *PINK1*, two patients carried deletions in exon 1 and 5, and the W90Xfs point mutation. Eighteen per cent of patients with age at onset ≤30 and 7.4% of patients from large dominant families carried pathogenic Mendelian gene mutations. Of all young-onset patients, 10 (3.3%) carried biallelic mutations in *PRKN* or *PINK1.* Across the whole cohort, 18 patients (0.9%) carried pathogenic *LRRK2 *mutations and one (0.05%) carried an *SNCA* duplication. There is a significant burden of *LRRK2 *G2019S in patients with both apparently sporadic and familial disease. In young-onset patients, dominant and recessive mutations were equally common. There were no differences in clinical features between *LRRK2* carriers and non-carriers. However, we did find that *PRKN *and *PINK1 *mutation carriers have distinctive clinical features compared to young-onset non-carriers, with more postural symptoms at diagnosis and less cognitive impairment, after adjusting for age and disease duration. This supports the idea that there is a distinct clinical profile of *PRKN *and *PINK1*-related Parkinson’s disease. We estimate that there are approaching 1000 patients with a known genetic aetiology in the UK Parkinson’s disease population. A small but significant number of patients carry causal variants in *LRRK2*, *SNCA*, *PRKN* and *PINK1* that could potentially be targeted by new therapies, such as *LRRK2* inhibitors.

## Teaser

In a large population-based study, Tan *et al.* show that pathogenic mutations are present in 1.4% of UK patients with Parkinson’s disease and are more common in those with early-onset or familial disease. Carriers of mutations in *PRKN* or *PINK1* have distinctive clinical features compared to non-carriers.

## Introduction

Parkinson’s disease is a progressive neurological condition which affects 140/100 000 individuals within the UK (Wickremaratchi *et al.*, 2009). It is caused by genetic mutations in *LRRK2*, *SNCA*, *PRKN *(also known as parkin or PARK2), and *PINK1* in up to 10% of patients (Lesage and Brice, 2012; Puschmann, 2013; Lubbe and Morris, 2014). These genetic factors also influence clinical features of the disease, such as age at onset (Clark *et al.*, 2007; Golub *et al.*, 2009; Lesage and Brice, 2012; Klebe *et al.*, 2013; Cilia *et al.*, 2016), motor features, presenting symptoms, disease progression (Davis *et al.*, 2016) and cognition (Alcalay *et al.*, 2012; Mata *et al.*, 2015; Crosiers *et al.*, 2016).

Many previous studies have focused on highly selected cohorts recruited from specialist clinics. This is likely to lead to bias both in estimates of frequency and clinical characteristics associated with specific genetic mutations.

To overcome these issues, we designed the ‘Tracking Parkinson’s study’, a large-scale population-based prospective cohort study of recently diagnosed and young-onset Parkinson’s disease patients in the UK. It is the largest single cohort study of genetic mutations in Parkinson’s disease and is relatively unbiased. Analysis of this cohort is important to: (i) develop more accurate estimates of genetic risk and the likelihood of a known genetic cause overall and in specific patient subgroups; (ii) estimate the likelihood of further high risk genes that have not yet been identified; and (iii) understand the contribution of Mendelian gene variation to the phenotype of Parkinson’s disease.

Several studies have examined the frequency of gene mutations in early-onset Parkinson’s disease patients (Alcalay *et al.*, 2010*a*; Kilarski *et al.*, 2012). However, some mutations, such as *LRRK2*, are also present at a significant rate in non-familial late-onset Parkinson’s disease patients (Clark *et al.*, 2006). Previous studies have also sometimes used single techniques such as partial Sanger sequencing, which are not able to detect copy number variation common in *PRKN* and less common point mutations. In our analysis, mutations were comprehensively identified using a range of different genetic screening methods, including whole-exome sequencing, multiplex ligation-dependent probe amplification (MLPA) and Sanger sequencing.

The aim of this study is to describe the frequency of pathogenic Mendelian gene variants in the general Parkinson’s disease population and in specific disease subgroups. In addition, we sought to understand the relationship between Mendelian mutations and clinical phenotype at presentation.

## Materials and methods

Patients were recruited to the Tracking Parkinson’s study from sites across the UK. Patients were required to have a clinical diagnosis of Parkinson’s disease fulfilling Queen Square Brain Bank criteria (Hughes *et al.*, 2001). This project was funded by Parkinson’s UK and supported by the National Institute for Health Research.

Patients with disease duration of <3.5 years at time of diagnosis were recruited as ‘recent onset’ participants. Patients with disease duration of >3.5 years at time of diagnosis and age at onset ≤ 50 years were recruited as ‘established young-onset’ participants. Patients were recruited regardless of ethnicity, including Jewish ethnicity. Full eligibility criteria, exclusion criteria and methods of recruitment have been described previously (Malek *et al.*, 2015). Importantly, unlike most studies of this type, patients were recruited irrespective of any prior information on genetic status.

Motor and non-motor features were assessed using standardized and validated scales, including the Movement Disorder Society Unified Parkinson’s Disease Rating Scale (MDS-UPDRS), Hoehn and Yahr stage and Montreal Cognitive Assessment (MoCA). Full details are provided in the Supplementary material.

Pathogenic mutations in the studied genes were defined according to MDSGene (http://www.mdsgene.org) (Lill *et al.*, 2016; Kasten *et al.*, 2018), and the Parkinson Disease Mutation Database (PDmutDB; http://www.molgen.vib-ua.be/Parkinson's diseaseMutDB/). Variants that did not meet pathogenicity criteria according to MDSGene (variants classified as ‘benign’) were not reported.

### Genetic analysis of Parkinson’s disease gene mutations

At study entry, blood samples were collected from every participant and DNA was extracted from an EDTA sample. We screened for mutations in *PRKN*, *PINK1 *and *GBA* with Sanger sequencing. As *GBA *is considered a risk gene for Parkinson’s rather than a pathogenic single gene cause, we reported the results of *GBA *sequencing separately (Malek *et al.*, 2018).

Whole exome sequencing was performed in a subset of young-onset and familial patients (*n = *489) (Supplementary material). Exome sequencing data was screened for pathogenic variants in *SNCA*, *LRR2K2*, *PRKN*, *PINK1*, *PARK7*/DJ1 and *VPS35.*

### Genotyping in young-onset and late-onset patients

Genotyping was carried out on 2106 patients with Parkinson’s disease for the *LRRK2* G2019S mutation using the ‘Kompetitive’ allele-specific polymerase chain reaction (KASP) assay (LGC Genomic Solutions).

We performed SNP array genotyping for 2116 samples. Samples were genotyped using the Illumina HumanCore Exome array supplemented with custom content, including over 27 000 custom variants that have been previously implicated in neurological, neurodegenerative and psychiatric conditions (Malek *et al.*, 2015). For imputation, genotypes were aligned to the 1000 Genomes Phase 3 v5 mixed population reference panel (Auton *et al.*, 2015) (build hg19/ GRCh37) and imputed using Minimac3 (Das *et al.*, 2016) on the Michigan Imputation Server (Supplementary material).

#### Genotyping in young-onset patients

Patients with age at onset ≤50 were screened for point mutations in *PRKN* and *PINK1* using Sanger sequencing (Supplementary Fig. 2). We also performed MLPA to detect and confirm copy number variation in *PRKN*, *PINK1*, *PARK7*/DJ1 and *SNCA. *MLPA was performed with the MRC Holland SALSA MLPA P051 Parkinson kit (version D1), according to the manufacturer’s instructions. Of 424 patients, 291 (68.7%) were successfully genotyped for *PRKN* and *PINK1* with both MLPA and Sanger sequencing. Eleven patients were screened for copy number variants using MLPA but were not Sanger sequenced. Exome sequencing was performed in 269 patients.

For our final phenotype-genotype analyses, we included young-onset patients if both MLPA and either Sanger or exome sequencing, had been completed. The combination of these methods was selected in order to detect both copy number variants and point mutations in *PRKN* and *PINK1.* In total, 302 patients with age at onset ≤50 were included for final analysis.

#### Genotyping in late-onset patients

Exome sequencing was performed in 219 late-onset patients with a positive family history of Parkinson’s disease and one patient with missing age at onset and a positive family history.

In late-onset patients with two or more additional family members affected by Parkinson’s disease, MLPA was performed in 65 of 74 (87.8%) patients.

For the final phenotype-genotype analyses, we included late-onset patients if either *LRRK2* KASP genotyping or exome sequencing had been successfully completed. In total, 1701 late-onset patients were included for final analysis, as well as two patients with missing age at onset.

In total, 2005 patients with Parkinson’s disease were included for final analysis (302 young-onset, 1701 late-onset, two missing age at onset).

### Mutations of uncertain pathogenicity

From the exome sequencing data, we report on the frequency of variants that have been previously reported in Parkinson’s disease or parkinsonism but whose pathogenicity is uncertain (Supplementary material and Supplementary Table 4).

This study was not designed to confirm pathogenicity of variants through segregation or comparison of allele frequencies in cases and controls. However, we report allele frequencies in our cohort from exome sequencing alongside allele frequencies in controls obtained from gnomAD (http://gnomad.broadinstitute.org/).

### Haplotype and relatedness analysis

Unimputed genotype data were used for pairwise identity-by-descent (IBD) analysis. Imputed genotype data were used for haplotype analysis. Individual haplotypes were constructed manually for mutation carriers. The markers used to construct haplotypes are detailed in the Supplementary material.

### Statistical analyses

Demographic characteristics were compared using *t*-tests, Fisher’s exact tests for proportions, or two-sample proportion tests. Linear regression was used for comparisons of demographic characteristics with covariate adjustment. To assess the association between clinical outcomes and genetic status, we used linear regressions of continuous scores against gene status (mutation positive or mutation negative) adjusting for age at assessment, disease duration at study entry, sex and LEDD. Hoehn and Yahr stage, MoCA subdomain and dystonia comparisons were conducted using ordered logistic regression. Motor subtype was analysed using multinomial logistic regression with the tremor dominant group as the comparator. All *P*-values were 2-tailed. We applied the Bonferroni correction for multiple testing for the number of independent tests in Tables 5 and 7. Statistical analysis was conducted using STATA (version 14, StataCorp, Texas, USA) and R (version 3.5.1).

### Prevalence estimates

We estimated the absolute numbers of Parkinson’s disease patients with a Mendelian genetic cause in the UK using the following approach in recent-onset patients only. Patients with established young-onset disease were not included for the prevalence estimate calculations. We used age-specific prevalence rates from a previous UK meta-analysis (Wickremaratchi *et al.*, 2009) and applied the rates to the Office of National Statistics Great Britain mid-2016 population estimates (Office for National Statistics, 2017) to derive an approximate number of all Parkinson’s disease patients. The age distribution of the Parkinson’s disease population (as a percentage) was used to standardize the rates of genetic Parkinson’s disease within our cohort (per 100 000). From this, we derived the new age-standardized rate of genetic Parkinson’s disease. We applied this age-standardization method because our over-sampling of young-onset cases had resulted in a non-representative age-distribution of patients. This new rate was then applied to the total Parkinson’s disease population to estimate the absolute number of patients with a Mendelian genetic cause in the UK population. It is important to note that, as we have derived the rates from our incident cases (excluded established young-onset cases), we have assumed that the rates are representative of all prevalent cases. This may not be true if these Mendelian forms of Parkinson’s disease are associated with better or worse survival, in which case our estimates will be either an under- or overestimate of the true numbers. Ninety-five per cent confidence intervals were calculated using the Poisson distribution.

### Data availability

The anonymized data from this study are available to researchers, to support other studies. Please apply via the Tracking Parkinson’s project coordinator (tracking-parkinsons@glasgow.ac.uk).

## Results

Table 1 shows the baseline demographics for participants that met Parkinson’s disease diagnostic criteria. Data are presented separately for three groups below, according to inclusion criteria for recruitment. Here we defined young-onset as patients with age at onset ≤50 (Malek *et al.*, 2015). Young-onset patients were separated into recent and established patients, as only the recent onset patients represent an incident, largely population-based cohort of Parkinson’s disease. For this reason, only recent onset patients were used to estimate the prevalence of genetic forms of Parkinson’s disease in the UK.

- Recent late-onset Parkinson’s disease patients (age at onset >50, disease duration ≤3.5 years at time of diagnosis);
- Recent young-onset Parkinson’s disease patients (age at onset ≤50, disease duration ≤3.5 years at time of diagnosis);
- Established young-onset Parkinson’s disease patients (age at onset ≤50, disease duration >3.5 years at time of diagnosis).

**Table 1:** Baseline demographics for all Parkinson’s disease patients with known age at onset

	**Recent, late-onset patients (AAO >50, ≤3.5 years from diagnosis) *n = *1799**	**Recent, young-onset patients (AAO ≤50, ≤3.5 years from diagnosis) *n = *197**	**Established young-onset patients (AAO ≤50, >3.5 years from diagnosis) *n = *227**	**Total *n = *2223**
Age at recruitment, years (SD)	69.3 (7.5)	48.8 (6.2)	54.5 (7.7)	66.0 (10.2)
Age at onset, years (SD)	66.4 (7.7)	43.7 (5.6)	41.1 (7.1)	61.8 (12.1)
Disease duration at diagnosis, years (SD)	1.3 (0.9)	1.4 (1.0)	11.4 (6.4)	2.4 (3.8)
Disease duration at entry, years (SD)	2.9 (2.1)	5.2 (6.6)	13.1 (7.4)	4.0 (4.6)
**Family history, *n* (%)**				
No family history	1442 (80.2)	145 (73.6)	166 (73.1)	1753 (78.9)
One additional affected family member	267 (14.8)	41 (20.8)	47 (20.7)	355 (16.0)
Two additional affected family members	59 (3.3)	8 (4.1)	8 (3.5)	75 (3.4)
Three additional affected family members	11 (0.6)	2 (1.0)	4 (1.8)	17 (0.8)
Four or more additional affected family members	4 (0.2)	0 (0.0)	1 (0.4)	5 (0.2)
Consistent with dominant inheritance	305 (17.0)	49 (24.9)	57 (25.1)	411 (18.5)
Consistent with recessive inheritance	36 (2.0)	2 (1.0)	3 (1.3)	41 (1.8)
**Consanguinity (%)**				
Non-consanguineous	1741 (96.8)	191 (97.0)	220 (96.9)	2152 (96.8)
Consanguineous	16 (0.9)	2 (1.0)	2 (0.9)	20 (0.9)
**Ethnicity (%)**				
White	1742 (96.8)	188 (95.4)	211 (93.0)	2141 (96.3)
Asian or Asian British	16 (0.9)	3 (1.5)	8 (3.5)	27 (1.2)
Black or Black British	10 (0.6)	3 (1.5)	2 (0.9)	15 (0.7)
Chinese	0 (0.0)	0 (0.0)	2 (0.9)	2 (0.1)
Mixed	4 (0.2)	0 (0.0)	0 (0.0)	4 (0.2)
Other	2 (0.1)	1 (0.5)	0 (0.0)	3 (0.1)
**Sex (%)**				
Male	1181 (65.7)	124 (62.9)	149 (65.6)	1454 (65.4)

AAO = age at onset.

Consistent with dominant inheritance = family members from multiple generations affected.

Consistent with recessive inheritance = family members only from the same generation affected.

Thirty-seven patients received a revised alternative diagnosis other than Parkinson’s disease or had conflicting dopamine transporter (DaT) scan results and were excluded from further analysis. On rare occasions, *LRRK2* mutations may be present in progressive supranuclear palsy or atypical parkinsonian patients (Sanchez-Contreras *et al.*, 2017; Vilas *et al.*, 2018); however, we did not identify any pathogenic mutations in these patients.

### Summary of genotyping

Supplementary Figs 1–5 show the number of patients that were genotyped with each method. The shaded boxes highlight the samples that were included for analysis. There were approximately 100 patients for which DNA was not available for genotyping (this varied between different methods). These patients were excluded from phenotype-genotype analyses.

For young-onset patients, we included samples for final analysis if MLPA had been completed, and either Sanger sequencing or exome sequencing or both had been successfully completed. In total, 302 patients with age at onset ≤50 were included for final analysis of *PRKN* and *PINK1.*

For late-onset patients, we included patients for final analysis if the samples had been genotyped with the *LRRK2* KASP assay for G2019S, and/or exome sequencing. In total, 1701 late-onset patients were included for final analysis, as well as two patients with missing age at onset.

In total, 2005 patients with Parkinson’s disease were included for final analysis (302 young-onset, 1701 late-onset, two missing age at onset).

### Summary of mutations identified

We identified 14 different pathogenic mutations in *LRRK2*, *SNCA*, *PRKN *and *PINK1* in 29 of 2005 patients [1.4%, 95% confidence interval (CI) 0.9–2.0%] (Tables 2 and 3). This estimate is conservative as not all samples were comprehensively tested, therefore the true mutation rate may be higher.

**Table 2:** Overall frequency of dominant gene mutation carriers in successfully genotyped patients

	Young onset *n* = 408	Late onset *n* = 1701	All *n* = 2003
***LRRK2***	9 (2.2%; 0.8–3.6%)	9 (0.5%; 0.2–0.9%)	18 (0.9%; 0.5–1.3%)
***SNCA***	0 (0%; 0.0–0.9%)	1 (0.06%; 0.01–0.3%)	1 (0.05%; 0.04–0.1%)
**All autosomal dominant (*LRRK2* and *SNCA*)**	9 (2.2%; 0.8–3.6%)	10 (0.6%; 0.2–1.0%)	19 (0.9%; 0.5–1.4%)

Percentages and 95% CIs are shown in parentheses.

**Table 3:** Overall frequency of biallelic recessive gene mutation carriers for known pathogenic variants in successfully genotyped young-onset patients (age at onset ≤50)

	Young onset *n* = 302
**PRKN**	
Homozygous	0 (0%; 0.0–0.1.3%)
Compound heterozygous	8 (2.6%; 0.8–4.5%)
**PINK1**	
Homozygous	1 (0.3%; 0.06–1.9%)
Compound heterozygous	1 (0.3%; 0.06–1.9%)
**All autosomal recessive (*PRKN* and *PINK1* biallelic mutations)**	10 (3.3%; 1.3–5.3%)

Percentages and 95% CIs are shown in parentheses.

Eighteen patients carried a mutation in *LRRK2*, one patient carried an *SNCA* mutation, eight patients carried biallelic *PRKN* mutations and two patients carried biallelic *PINK1* mutations. No patients were found carrying pathogenic mutations in *VPS35* or *PARK7* (DJ1). No patient carried pathogenic mutations in more than one gene. Three patients carried the *LRRK2* G2019S mutation and additionally one or more mutations in *GBA* (p.E326K and p.P122H). The mean age at onset for patients carrying mutations in both *LRRK2* and *GBA* was 43.2 years [standard deviation (SD) = 5.1], compared to an age at onset of 56.5 years (SD = 12.9) for *LRRK2* mutation carriers without *GBA *mutations. Pathogenic carriers are shown in Supplementary Table 1 and the list of unique mutations are shown in Supplementary Table 2.

We identified nine patients carrying single heterozygous pathogenic mutations in *PRKN* and *PINK1* (Supplementary Table 3). Previous analysis of this cohort showed no differences between carriers of single heterozygous *PRKN* mutations (including mutations of uncertain pathogenicity) and non-carriers other than in olfaction (Malek *et al.*, 2016), therefore patients with single heterozygous mutations in recessive genes were analysed as non-carriers.

One patient carried three pathogenic mutations in *PRKN* (Supplementary Table 1).

Mutations were common in patients with very young onset and patients with multiple family members also affected by Parkinson’s disease. Of Parkinson’s disease patients with onset ≤30, 18.8% (3/16; 95% CI 6.6–43.0%) carried pathogenic mutations. In young-onset patients, 18.2% (4/22; 95% CI 7.3–38.5%) of patients with two or more additional affected family members carried pathogenic mutations. In late-onset patients, 4.2% (3/72; 95% CI 1.4–11.5%) of patients with two or more additional affected family members carried pathogenic mutations.

Notably, the *LRRK2* G2019S mutation was more common in young-onset patients (2.2%, 9/408; 95% CI 0.7–3.6%) than in later-onset patients (0.4%, 7/1701; 95% CI 0.1–0.7%), *P = *0.001 [Fisher’s exact test, odds ratio (OR) = 5.5, 95% CI 1.8–17.3]. In addition, young-onset patients were equally likely to have recessive (2.5%, 10/408) and dominant pathogenic mutations (2.2%, 9/408).

Pathogenic mutations were only identified in patients reporting ‘White’ ethnicity (*n = *2005 genotyped).

IBD analysis was conducted based on 25 781 single nucleotide polymorphisms (SNPs) in linkage equilibrium. This showed that none of the mutation carriers were related to each other (pi-hat <0.1 for all, indicating no closer relations than third-degree relatives).

Constructed haplotypes and the results of haplotype analysis are shown in Supplementary Figs 6–9).

#### LRRK2

We identified 18 patients carrying heterozygous *LRRK2* mutations, either G2019S (*n = *16) or R1441C (*n = *2). 55.6% (10/18) carriers reported a positive family history of Parkinson’s disease.

Both *LRRK2* R1441C carriers reported a family history of Parkinson’s disease. As we only screened for the R1441C mutation through exome sequencing in familial and/or young-onset patients, our results for R1441C cannot be used to compare familial versus non-familial patients.

We only included *LRRK2* G2019S mutation carriers for our analysis of family history. G2019S mutations were more common among patients with a positive family history (1.9%, 95% CI 0.5–3.1%) than patients without a family history of Parkinson’s disease (0.5%, 95% CI 0.1–0.8%), *P = *0.009 (Fisher’s exact test, OR = 3.9, 95% CI 1.3–11.8). However, within the G2019S carriers, 50% had a positive family history and 50% did not have a family history of Parkinson’s (50%, 95% CI 25.5–74.5%).

*LRRK2* mutation carriers (G2019S and R1441C carriers together) had an earlier mean age at onset (54.3 years, 95% CI 47.9–60.7) compared to non-carriers (61.7 years, 95% CI 61.2–62.2; *P = *0.01). Age at onset for *LRRK2* carriers ranged from 35.2 to 78.7 years. *LRRK2* mutations were more frequent in young-onset (2.2%, 95% CI 1.0–4.2%) compared to late-onset patients (0.5%, 95% CI 0.2–1.0%), *P = *0.003 (Fisher’s exact test, OR = 4.2, 95% CI = 1.5–12.1).

Clinical features of *LRRK2* carriers compared to non-carriers are presented in Table 5 (excluding patients with recessive gene mutations). We did not include the *SNCA *carrier in this analysis given that previous literature suggests that *LRRK2* and *SNCA *mutation carriers have different clinical features (Trinh *et al.*, 2018). We did not find any differences in clinical features between *LRRK2 *carriers and non-carriers.

**Table 4:** Rate of known dominant pathogenic mutations based on clinical presentation

	*LRRK2 n = *18	*SNCA n = *1	Rate of all pathogenic dominant mutations
**Age at onset (%)**			
≤20 years, *n = *4	0/4 (0)	0/4 (0)	0/4 (0)
≤30 years, *n = *18	0/18 (0)	0/18 (0)	0/18 (0)
≤40 years, *n = *118	2/118 (1.7)	0/118 (0)	2/118 (1.7)
≤50 years, *n = *408	9/408 (2.2)	0/408 (0)	9/408 (2.2)
≤60 years, *n = *784	10/784 (1.3)	1/784 (0.1)	11/784 (1.4)
≤70 years, *n = *1552	17/1552 (1.1)	1/1552 (0.06)	18/1552 (1.2)
≤80 years, *n = *2050	18/2050 (0.9)	1/2050 (0.05)	19/2050 (0.9)
All, *n = *2109	18/2109 (0.9)	1/2109 (0.05)	19/2109 (0.9)
**Mean age of onset in years (SD)**	54.3 (12.9)	-	54.1 (12.6)
**Family history (%)**			
No other family members affected	8/1658 (0.5)	0/1658 (0)	8/1658 (0.5)
One other family member affected	7/344 (2.0)	0/344 (0)	7/344 (2.0)
Two other family members affected	1/72 (1.4)	1/72 (1.4)	2/72 (2.8)
Three other family members affected	2/17 (11.8)	0/17 (0)	2/17 (11.8)
Four or more family members affected	0/5 (0)	0/5 (0)	0/5 (0)

**Table 5:** Comparison of motor features, fluctuations and non-motor features by *LRRK2 *mutation status (*LRRK2* carriers versus non-carriers)

Variable	Mutation negative *n = *2082	LRRK2 positive *n = *18	Beta (95% CI) LRRK2 carriers versus non-carriers	*P*-value^a^
Age at entry, years	66.0 (10.1)	60.1 (10.4)	−5.2 (−9.9, −0.5)	**0.030** **^b^**
Age at onset, years	61.8 (11.9)	54.3 (12.9)	−5.2 (−9.9, −0.5)	**0.030** **^b^**
Disease duration, years	4.0 (4.4)	5.2 (4.5)	0.7 (−1.3, 2.8)	0.482^c^
Delay to diagnosis (time from symptom onset to diagnosis), years	1.8 (2.9)	1.5 (1.3)	−0.4 (−1.8, 1.0)	0.580^c^
**Motor features**				
MDS-UPDRS III total score	23.4 (12.7)	28.6 (15.2)	6.7 (0.1, 13.3)	0.047
Severity score MDS-UPDRS-III/years from symptom onset	10.4 (11.8)	9.4 (7.3)	0.6 (−5.7, 6.8)	0.862^d^
Upper limb score, max 56	10.7 (6.3)	12.1 (6.3)	2.1 (−0.9, 5.1)	0.163
Lower limb score, max 32	5.1 (3.9)	6.8 (5.5)	1.7 (−0.2, 3.6)	0.085
Gait and freezing, max 8	1.1 (1.1)	1.6 (1.7)	0.4 (−0.1, 0.9)	0.097
Hoehn and Yahr stage			0.3 (−0.7, 1.2)	0.595
0–1.5 (%)	950 (46.0)	7 (38.9)		
2 or 2.5 (%)	957 (46.3)	10 (55.6)		
3+ (%)	160 (7.7)	1 (5.6)		
**Symptoms present at diagnosis (%)**				
Tremor	1499/2017 (74.3)	13/18 (72.2)	0.3 (−0.8, 1.6)	0.586
Rigidity	1410/1925 (73.2)	13/18 (72.2)	−0.08 (−1.2, 1.2)	0.891
Bradykinesia	1554/1966 (79.0)	12/18 (66.7)	−0.8 (−1.8, 0.3)	0.121
Postural problems	363/1898 (19.1)	4/18 (22.2)	0.009 (−1.5, 1.2)	0.989
Other	456/1827 (25.0)	4/16 (25 )	0.2 (−1.1, 1.3)	0.731
Motor subtype				
Tremor dominant	835/1892 (44.1)	7/17 (41.2)		
Non-tremor dominant (PIGD)	813/1892 (43.0)	10/17 (58.8)	−2.8 (−0.5, 1.8)	0.246
Mixed	244/1892 (12.9)	0/17 (0)	−8.7 (NA)^e^	NA^e^
**Motor complications**				
MDS-UPDRS-IV total score	1.3 (2.8)	2.8 (3.3)	0.1 (−0.9, 1.2)	0.794
Dyskinesias (MDS-UPDRS IV part 1 and 2 sum, max 8)	0.3 (1.0)	0.4 (0.9)	−0.2 (−0.5, 0.1)	0.259
Fluctuations (MDS-UPDRS IV part 3–5 sum, max 12)	0.9 (1.9)	2.1 (2.6)	0.3 (−0.4, 1.1)	0.408
Dystonia, max 4	0.2 (0.6)	0.3 (0.6)	0.01 (−0.2, 0.3)	0.915
**Non-motor features**				
Cognition: total MoCA score	25.2 (3.5)	25.4 (3.2)	−0.2 (−1.9, 1.4)	0.761
Visuospatial, max 5	4.3 (1.1)	4.2 (1.2)	−0.2 (−0.7, 0.3)	0.359
Naming, max 3	2.9 (0.3)	2.9 (0.3)	−0.05 (−0.2, 0.1)	0.535
Attention, max 6	5.2 (1.0)	5.3 (0.8)	0.1 (−0.4, 0.6)	0.690
Language, max 3	2.4 (0.8)	2.4 (0.7)	−0.03 (−0.4, 0.3)	0.865
Abstraction, max 2	1.6 (0.6)	1.7 (0.7)	0.003 (−0.3, 0.3)	0.983
Recall, max 5	2.7 (1.6)	2.9 (1.8)	0.05 (−0.7, 0.8)	0.898
Orientation, max 6	5.8 (0.5)	5.8 (0.5)	−0.03 (−0.2, 0.2)	0.756
LADS Anxiety score, max 18)	4.5 (3.8)	5.8 (3.8)	0.9 (−0.8, 2.6)	0.287
LADS Depression score, max 18	4.5 (3.3)	5.1 (3.3)	0.3 (−1.2, 1.8)	0.706
Sleep disturbance, ESS score	7.1 (4.8)	9.7 (6.8)	1.6 (−0.7, 3.8)	0.173
RBDSQ scale score	4.8 (3.2)	6.4 (3.5)	1.0 (−0.5, 2.5)	0.191
Autonomic function: SCOPA total score	9.3 (5.8)	10.8 (6.4)	2.6 (−1.1, 6.3)	0.170

Patients carrying biallelic recessive mutations and one patient carrying a *SNCA *mutation were excluded from analyses. Scores in the first two columns are means (SD), except for Hoehn and Yahr stage, symptoms present at diagnosis and motor subtype which are shown as *n* or proportions (%). Increasing scores and increasing beta values for motor and non-motor variables are associated with worse symptoms, with the exception of the MoCA test scores. Increasing scores and increasing beta values for the MoCA test are associated with better cognition.

ESS = Epworth Sleep Scale; LADS = Leeds Anxiety and Depression Scale; MDS-UPDRS = Movement Disorder Society Unified Parkinson’s Disease Rating Scale; MoCA = Montreal Cognitive Assessment; NA = not assessed; PIGD = postural instability gait difficulty; RBDSQ = Rapid Eye Movement Sleep Behaviour Disorder Screening Questionnaire; SCOPA = Scales for Outcomes in Parkinson’s disease.

^a^ *P*-value of clinical features of *LRRK2* carriers together compared to non-carriers, excluding patients with recessive gene mutations and one patient with *SNCA *mutation. Adjusting for age at entry, gender, disease duration at entry/assessment and LEDD total, unless otherwise specified.

^b^Adjusting for gender and disease duration at entry.

^c^Adjusting for gender and age at entry.

^d^Adjusting for age, gender and LEDD total.

^e^Insufficient count to fit model.

#### SNCA

*SNCA* copy number variants were screened with MLPA in 65 patients with familial Parkinson’s disease with two or more family members affected. One patient (1.5%) carrying a heterozygous whole gene duplication was identified, who reported two additional family members affected by Parkinson’s disease. We were unable to compare the clinical features of *SNCA* carriers to non-carriers given that only one *SNCA* carrier was identified.

### Young-onset patients

We identified 19/302 (6.3%) young-onset patients carrying pathogenic mutations in both dominant and recessive genes. Here we defined young-onset as patients with age of onset ≤50. The proportions of mutation carriers by age at onset and family history are presented in Table 6. Recessive gene mutation carriers had an earlier mean age at onset (32.7 years) compared to non-carriers (41.1 years), *P* < 0.001, excluding dominant mutation carriers.

**Table 6:** Cumulative rate of pathogenic mutations based on clinical presentation in successfully genotyped young-onset Parkinson’s disease patients (age at onset ≤ 50), *n = *302

	*PINK1* (biallelic) *n = *2	*PRKN* (biallelic) *n = *8	All recessive gene mutations *n = *10
**Age at onset (%)**			
≤20 years, *n = *4	0/4 (0)	2/4 (50)	2/4 (50)
≤30 years, *n = *18	0/16 (0)	3/16 (18.8)	3/16 (18.8)
≤40 years, *n = *118	1/110 (0.9)	6/110 (5.5)	7/110 (6.4)
≤50 years, *n = *408	2/302 (0.7)	8/302 (2.6)	10/302 (3.3)
**Mean age of onset in years (SD)**	42.3 (5.5)	30.3 (11.5)	-
**Family history (%)**			
No other family members affected	1/213 (0.5)	4/213 (1.9)	5/213 (2.3)
One other family member affected	1/67 (1.5)	1/67 (1.5)	2/67 (3.0)
Two other family members affected	0/15 (0)	3/15 (20)	3/15 (20)
Three other family members affected	0/6 (0)	0/6 (0)	0/6 (0)
Four or more other family members affected	0/1 (0)	0/1 (0)	0/1 (0)

When considering all young-onset mutation carriers (*PRKN*, *PINK1*, *LRRK2* and *SNCA*), the mean age at onset was also younger than non-carriers (37.5 versus 41.1 years; *P = *0.02). Mutations were more frequent in patients with a positive family history (11.0%) than in patients with no family history of Parkinson’s disease (4.2%), *P = *0.04 (Fisher’s exact test, OR = 2.8, 95% CI 1.0–8.1).

#### PRKN

Of all young-onset patients that were successfully genotyped for *PRKN*, biallelic pathogenic *PRKN* mutations were present in 2.6% (8/302, 95% CI 0.8–4.4%). No *PRKN* carriers had homozygous mutations; all mutations were present in compound heterozygous state.

*PRKN* mutations were present in 20% (3/15, 95% CI 7.0–45.2%) of young-onset patients with two additional family members affected by Parkinson’s disease. However, there was no significant difference in the frequency of mutations in early young-patients with a positive family history (4.2%, 95% CI 0.2–8.4%) and without a family history of Parkinson’s disease (1.9%, 95% 0.05–3.7%), *P* > 0.2 (Fisher’s exact test, OR = 2.3, 95% CI 0.4–12.9). Young-onset patients from large Parkinson’s disease families (two or more additional family members affected) were more likely to carry a *PRKN* mutation (13.6%) than young-onset patients with one or no additional family members affected (1.6%), *P = *0.01 (Fisher’s exact test, OR = 8.5, 95% CI 1.2–47.9).

The clinical features of *PRKN* and *PINK1* mutation carriers compared to young-onset non-carriers are presented in Table 7. *PRKN* carriers had younger age at onset than young-onset patients with *LRRK2* mutations (42.9 years, 95% CI 39.3–46.6), *P = *0.009. There was no difference in age at onset of *PRKN* and *PINK1* carriers, *P* > 0.2.

#### PINK1

Biallelic *PINK1* mutations were present in 0.7% (2/302, 95% CI 0.2–2.4%) of all screened young-onset patients. Mutations were present in 1.1% (1/89) of young-onset patients with a positive family history and 0.5% (1/213) of patients with no family history of Parkinson’s disease. Mutations were not more frequent in patients with a positive family history, *P = *0.50 (Fisher’s exact test, OR = 2.4, 95% CI 0.03–189.7).

*PRKN *and *PINK1 *mutation carriers had earlier age at onset than other young-onset non-carriers, adjusting for gender and disease duration (Table 7). They also had longer disease duration than non-carriers, adjusting for age at entry and gender (Table 7).

**Table 7:** Comparison of motor features, fluctuations and non-motor features of young-onset patients by recessive gene status (*PRKN* and *PINK1* carriers versus non-carriers), excluding patients carrying dominant gene mutations

Variable	Mutation-negative	Mutation-positive (biallelic)			Beta (95% CI)	*P*-value^a^
	*n = *292	Total, *n = *10	*PRKN, n = *8	*PINK1, n = *2	Carriers versus non-carriers	
Age at entry, years	51.9 (8.1)	50.9 (11.1)	51.8 (12.2)	47.5 (5.9)	−7.0 (−10.9, −3.1)	**0.001** **^b^**
Age at onset, years	41.1 (6.2)	32.7 (11.5)	30.3 (11.5)	42.3 (5.5)	−7.0 (−10.9, −3.1)	**0.001** **^b^**
Disease duration, years	10.4 (7.6)	18.2 (14.4)	21.9 (14.4)	5.2 (0.4)	8.9 (5.0, 12.7)	**<0.001** **^c^**
Delay to diagnosis, years	2.4 (4.2)	4.5 (4.1)	5.2 (4.4)	2.2 (0.1)	2.2 (−0.6, 5.1)	0.123^c^
**Motor features**							
MDS-UPDRS-III total score	26.1 (14.9)	29.0 (24.0)	33.0 (23.6)	5.0 (*n = *1)	−3.3 (−14.4, 7.8)	0.564
Severity score MDS-UPDRS-III/years from symptom onset	4.1 (6.8)	2.4 (2.9)	2.7 (3.1)	0.9 (*n = *1)	−2.5 (−7.7, 2.8)	0.356^d^
Upper limb score, max 56	11.6 (6.7)	13.9 (8.8)	15.3 (8.7)	8.5 (9.2)	−1.1 (−5.5, 3.3)	0.621
Lower limb score, max 32	6.2 (4.4)	7.7 (5.6)	8.5 (6.0)	4.5 (3.5)	−0.1 (−3.1, 3.0)	0.973
Gait and freezing, max 8	1.6 (1.5)	3.2 (1.9)	3.6 (1.7)	1.5 (2.2)	1.1 (0.03, 2.1)	**0.043**
Hoehn and Yahr stage					1.8 (0.1, 3.6)	0.049
0–1.5 (%)	107 (36.7)	1 (11.1)	1 (12.5)	0 (0)	-	-
2 or 2.5 (%)	140 (48.1)	4 (44.4)	3 (37.5)	1 (100)	-	-
3+ (%)	44 (15.1)	4 (44.4)	4 (50)	0 (0)	-	-
**Symptoms present at diagnosis (%)**							
Tremor	188/263 (71.5)	7/10 (70.0)	6/8 (75.0)	1/2 (50.0)	−0.9 (−2.4 0.8)	0.275
Rigidity	204/255 (80)	8/9 (88.9)	6/7 (85.7)	2/2 (100)	0.7 (−1.2, 3.7)	0.561
Bradykinesia	209/257 (81.3)	9/10 (90.0)	7/8 (87.5)	2/2 (100)	15.1 (−55.4, NA)	0.986
Postural problems	39/252 (15.5)	6/9 (66.7)	6/7 (85.7)	0/2 (0)	2.3 (0.7, 4.0)	**0.005**
Other	54/229 (23.6)	3/9 (33.3)	3/7 (42.9)	0/2 (0)	0.4 (−1.6, 2.0)	0.684
**Motor subtype (%)**							
Tremor dominant	79/257 (30.7)	2/8 (25.0)	1/6 (16.7)	1/2 (50)	-	-
Non-tremor dominant (PIGD)	150/257 (58.4)	6/8 (75.0)	5/6 (83.3)	1/2 (50)	0.4 (−1.4, 2.3)	0.646
Mixed/indeterminate	28/257 (10.9)	0/8 (0)	0/6 (0)	0/2 (0)	-9.5 (NA, NA)	>0.1
**Motor complications**							
MDS-UPDRS-IV total score	5.0 (4.9)	6.2 (5.7)	6.1 (6.3)	6.5 (3.5)	2.3 (−0.5, 4.5)	0.105
Dyskinesias, presence and severity; max 8	1.3 (1.9)	2.3 (2.5)	2.1 (2.8)	3.0 (1.4)	1.2 (0.03, 2.3)	**0.04**
Fluctuations, max 12	3.0 (2.9)	3.3 (4.0)	3.4 (4.3)	3.0 (4.2)	0.9 (−0.8, 2.6)	0.309
Dystonia, max 4	0.7 (1.1)	0.6 (1.3)	0.6 (1.4)	0.5 (0.7)	0.1 (−0.7, 0.8)	0.891
**Non-motor features**							
Cognition: total MoCA score, max 30	25.6 (3.6)	27.6 (2.2)	27.4 (2.3)	29.0 (*n = *1)	3.0 (0.8, 5.2)	**0.007**
Visuospatial, max 5	4.4 (1.1)	4.3 (0.5)	4.4 (0.5)	4.0 (*n = *1)	0.07 (−0.6, 0.8)	0.847
Naming, max 3	2.9 (0.3)	2.9 (0.3)	2.9 (0.4)	3.0 (0.0)	0.08 (−1.2, 0.3)	0.441
Attention, max 6	5.1 (1.0)	5.6 (0.5)	5.5 (0.5)	6.0 (0.0)	0.9 (0.3, 1.6)	**0.004**
Language, max 3	2.5 (0.7)	2.3 (0.8)	2.4 (0.7)	2.0 (1.4)	−0.07 (−0.5, 0.4)	0.767
Abstraction, max 2	1.7 (0.6)	1.6 (0.7)	1.6 (0.7)	1.5 (0.7)	0.09 (−0.4, 0.5)	0.704
Recall, max 5	3.1 (1.6)	4.2 (1.3)	4.3 (1.4)	4.0 (1.4)	0.9 (−0.2, 2.0)	0.116
Orientation, max 6	5.7 (0.7)	6.0 (0.0)	6.0 (0.0)	6.0 (0.0)	0.3 (−0.08, 0.6)	0.131
LADS Anxiety score, max 18	6.6 (4.2)	6.1 (2.6)	6.3 (2.8)	5.5 (2.1)	−0.4 (−3.3, 2.4)	0.763
LADS Depression score, max 18	5.8 (3.5)	5.8 (2.3)	6.4 (1.8)	3.5 (3.5)	−0.2 (−2.7, 2.4)	0.901
Sleep disturbance, ESS score	9.0 (5.7)	8.5 (7.6)	9.5 (8.3)	4.5 (2.1)	−0.1 (−4.2, 4.0)	0.961
RBDSQ scale score	5.8 (3.4)	4.3 (2.5)	4.4 (2.8)	4.0 (0.0)	−1.2 (−3.6, 1.1)	0.307
Autonomic function: SCOPA total score	10.8 (6.9)	12.3 (7.4)	9.5 (4.8)	20.5 (9.2)	0.1 (−5.0, 5.3)	0.959

Scores in the first four columns are mean (SD), except for Hoehn and Yahr stage, symptoms present at diagnosis and motor subtype which are shown as *n* or proportions (%). Increasing values and increasing betas for motor and non-motor variables are associated with worse symptoms, with the exception of the MoCA test scores. Increasing values and increasing betas for the MoCA test are associated with better cognition. Cells with only a single case are indicated with (*n* = 1).

^a^ *P*-value of clinical features of *PRKN* and *PINK1* carriers together compared to non-carriers, excluding patients with dominant gene mutations. Adjusting for age at entry, gender, disease duration at entry/assessment and LEDD total, unless otherwise specified.

^b^Adjusting for gender and disease duration at entry.

^c^Adjusting for gender and age at entry.

^d^Adjusting for age, gender and LEDD total.

MDS-UPDRS = Movement Disorder Society Unified Parkinson’s Disease Rating Scale; PIGD = postural instability gait difficulty; MoCA = Montreal Cognitive Assessment; LADS = Leeds Anxiety and Depression Scale; ESS = Epworth Sleep Scale; RBDSQ = Rapid Eye Movement Sleep Behaviour Disorder Screening Questionnaire; SCOPA = Scales for Outcomes in Parkinson’s disease.

*PRKN *and *PINK1 *mutation carriers also reported more postural problems at diagnosis than non-carriers and tended to report a higher rate of dyskinesias, after adjusting for age at entry, gender, disease duration and LEDD total, although this did not survive correction for multiple testing. They also tended to have more gait and freezing problems at assessment, after adjusting for age, gender, disease duration and LEDD total (*P = *0.021), although this was not significant after correction for multiple testing.

Finally, *PRKN *and *PINK1 *carriers had better cognition than non-carriers as assessed by the MoCA, even after adjusting for age, gender, disease duration and LEDD (*P = *0.007). This appears to be driven by better performance in the attention subdomain (*P = *0.004) though one must be cautious in interpreting the subdomains as they may be overly simplistic.

### Genes of unconfirmed pathogenicity for Parkinson’s disease

Patients carrying variants of unconfirmed pathogenicity and risk variants for Parkinson’s disease identified from exome sequencing are reported in Supplementary Table 4, including variants in *GIGYF2*, *CHDCHD2.* These variants were detected in cases, as previously described, but also almost all occur in the control population and were not included as pathogenic variants in our analysis.

We found comparable mutation/variant frequencies in our cohort compared to controls, with the exception of well-validated risk variants, such as *MAPT *(Martin *et al.*, 2001; Kwok *et al.*, 2004). We did not find any patients carrying previously reported mutations in *EIF4G1*, *DNAJC6*, *FBXO7 *and *PLA2G6.* Further case-control studies are needed to determine the role of variants in *SNCAIP*, *UCHL1* and other genes where we found small differences in allele frequencies from control frequencies, however these variants are unlikely to be pathogenic Mendelian mutations.

### Prevalence

In the recent onset cohort (both young-onset and late-onset), the frequency of pathogenic mutations was 1.0% (17/1787). This is a large-scale cohort unselected for age at onset, family history and genetic status. From this, we can estimate the frequency of pathogenic mutations in the general UK Parkinson’s disease population. The crude prevalence rate of genetic forms of Parkinson’s disease is 951 per 100 000 (95% CI 892–1013, using Poisson distribution). Age specific rates are presented in Table 8. The age-standardized rate of genetic forms of Parkinson’s disease was 708 per 100 000 (95% CI 657–762 per 100 000), standardized to the mid-2016 Great Britain population. This provides an estimate of ∼725 genetic Parkinson’s disease patients in a total of 102 403 patients in the UK currently living, using estimates from a meta-analysis (Wickremaratchi *et al.*, 2009) and the Office of National Statistics Great Britain population estimates for mid-2016 (Office for National Statistics, 2017) assuming these genes do not impact on survival (see ‘Materials and methods’ section). A recent report from Parkinson’s UK using primary care diagnosis estimated a larger number of Parkinson’s disease patients in the UK (145 519) in 2018 (Parkinson’s UK, 2017). If this figure is more accurate, then the number of genetic Parkinson’s disease cases would be larger (estimated at 1030).

**Table 8:** Age specific and crude prevalence rate of genetic forms of Parkinson’s disease, using data from recent-onset patients only

**Age**	**Parkinson’s disease genetic patients in cohort**	**Total number of Parkinson’s disease patients in cohort (screened)**	**Age specific rates per 100 000 Parkinson’s disease patients**
0–29	0	0	0
30–39	1	11	9091
40–49	4	58	6897
50–59	4	219	1826
60–69	5	728	687
70–79	2	633	316
≥80	1	138	725
**Total**	**17**	**1787**	-
Crude prevalence per 100 000 Parkinson’s disease patients	951 (525–1442)	-	-
Age-adjusted prevalence per 100 000 Parkinson’s disease patients^a^	708 (612–713)	-	-

^a^Age distribution derived from age-specific Parkinson’s disease rates (Wickremaratchi *et al.*, 2009) applied to the UK mid-2016 population estimates (Office for National Statistics, 2017).

## Discussion

This study represents the largest study examining the frequency of known Parkinson’s disease gene mutations. We report an overall frequency of mutations of 1.4% (29/2005), across both young-onset and late-onset patients. In combination with *GBA* gene analysis in the same cohort (Malek *et al.*, 2018), our results suggest that up to 10% of Parkinson’s disease patients carry a genetic variant that could potentially be targeted by new drug therapies. For instance, G2019S and other mutations in the *LRRK2 *gene have been shown to increase kinase activity, and *LRRK2* kinase inhibitors that counteract this activity are currently being tested in phase 1 clinical trials as a potential therapeutic target for Parkinson’s disease (reviewed in Atashrazm and Dzamko, 2016; Taymans and Greggio, 2016; Alessi and Sammler, 2018).

The strengths of this study lie in the relatively unbiased, population-based patient ascertainment. This increases the generalizability of our findings, specifically the prevalence estimates of Parkinson’s disease patients carrying pathogenic mutations based on the incident recent-onset cohort. A further strength of this study is inclusion of both early and late-onset patients, where previous genetic studies have focused on young-onset patients.

First, this has enabled us to more accurately estimate the prevalence of mutations in the general Parkinson’s disease UK population, assuming there are no survival effects, rather than just in the subset of young-onset patients. We show clearly that *LRRK2* mutations are present at a significant rate in patients with onset under 50 years (2.2%), and that *SNCA* mutations are present in 1.5% of patients with a strong family history of Parkinson’s disease (two or more additional family members affected).

Second, our findings suggest that there may be other high-risk genes that have not yet been identified. In particular, further efforts in gene discovery can focus on the substantial proportion of patients with very early onset or who have a large family history in which no known pathogenic mutations have been identified.

Third, our findings have implications for genetic testing. Although further work is needed to confirm some results, our data suggest that *LRRK2* mutations are common in young-onset Parkinson’s disease (2.2%) and should be more regularly tested with appropriate genetic counselling. Additionally, our results highlight the importance of systematically screening for copy number variants in *PRKN*, *PINK1* and *SNCA*, as these are not infrequent and may be missed with sequencing methods such as exome sequencing.

Finally, we show there are systematic clinical differences between recessive gene mutation carriers compared to young-onset non-carriers. *PRKN* and *PINK1* carriers have more postural problems at diagnosis and better cognition than other young-onset patients, even after adjusting for age, disease duration, gender and LEDD.

### *LRRK2* and *SNCA*

Mutations in *LRRK2* (PARK8, dardarin) were first identified in autosomal dominant, mostly late-onset families with Parkinson’s disease (Funayama *et al.*, 2002; Paisán-Ruíz *et al.*, 2004; Zimprich *et al.*, 2004). The reported frequency of *LRRK2* mutations varies widely; mutations are more common in familial Parkinson’s disease (5–6%) (Di Fonzo *et al.*, 2005; Nichols *et al.*, 2005) than in sporadic disease (∼1%) (Gilks *et al.*, 2005; Hernandez *et al.*, 2005). However, the frequency of mutations also differs according to population, and the G2019S mutation may be more common in Southern Europe than in Northern Europe (Bonifati, 2007). The rate of mutations is particularly high in Ashkenazi Jewish (up to 28%) and North African patients (up to 41%) (Lesage *et al.*, 2005, 2006; Ozelius *et al.*, 2006; Williams-Gray *et al.*, 2006; Healy *et al.*, 2008; Puschmann, 2013). We found that *LRRK2* mutations were present at a rate of 0.9% overall, most commonly the G2019S mutation (0.8%). Our findings are comparable with a previous community-based cohort in the UK (Williams-Gray *et al.*, 2006) and other Caucasian North American and UK cohorts with estimates between 0.4 and 1.7% (Deng *et al.*, 2005; Farrer *et al.*, 2005; Hernandez *et al.*, 2005; Zabetian *et al.*, 2005). Our results are also in accordance with a combined analysis of previous G2019S studies which estimated a mean prevalence of 0.9%, although this was across different populations (Williams-Gray *et al.*, 2006).

R1441C mutations were present in 0.4% of young-onset and familial patients. This is in keeping with other studies showing the rarity of *LRRK2* R1441C mutations in Caucasian populations, with previous studies reporting frequencies between 0% and 0.3% (Zabetian *et al.*, 2005; Pankratz *et al.*, 2006; Möller *et al.*, 2008). To our knowledge, this study is the first to systematically screen and report on the prevalence of R1441C mutations in young-onset and/or familial Parkinson’s disease in the UK.

Almost half of our *LRRK2* carriers did not report a family history of Parkinson’s disease. Although the first reports of *LRRK2* mutations were in families with multiple affected members, later studies have shown that a large proportion of *LRRK2* carriers do not have other family members affected by Parkinson’s disease (Gilks *et al.*, 2005; Ozelius *et al.*, 2006). This is likely due to the reduced penetrance of *LRRK2* mutations. The penetrance of both the G2019S and R1441C mutations is incomplete (24% to 42% up to age 80 for G2019S), strongly age-dependent and increases in a linear fashion (Clark *et al.*, 2006; Ozelius *et al.*, 2006; Healy *et al.*, 2008; Lee *et al.*, 2017). As the population ages, it is likely that increasing numbers of *LRRK2* relatives will develop Parkinson’s disease as a result of *LRRK2* mutations, and the prevalence of this form of Parkinson’s disease will increase in the UK.

As reported in some previous studies (Di Fonzo *et al.*, 2005; Gilks *et al.*, 2005; Kay *et al.*, 2006; Haugarvoll *et al.*, 2008), we found that *LRRK2* carriers presented with a range of age at onsets (35 to 79 years). *LRRK2* mutations were also more common in young-onset patients (2.2%) than in late-onset patients (0.5%). However, a combined analysis of all studies in MDSGene showed that the majority (94%) of *LRRK2* carriers have late age at onset (Trinh *et al.*, 2018). Our findings do not support this pattern and further work must be done to clarify this. It may be that studies included in MDSGene were more likely to screen late-onset patients and not young-onset patients for *LRRK2.* This is difficult to assess as MDSGene only compares characteristics of mutation carriers and not non-carriers. Our findings may have implications for genetic testing where, in the UK, *LRRK2* testing is recommended for late-onset patients with a family history of Parkinson’s disease. We suggest that *LRRK2* should be tested more frequently in young-onset patients, even those without a family history of Parkinson’s disease; however, additional studies in both young-onset and late-onset patients are needed.

We report two distinct G2019S haplotypes, in keeping with previous studies showing the mutation has been found in three major haplotypes. Haplotype 1 is the most common, present in European and North American populations of European, Arab and Jewish origin (Goldwurm *et al.*, 2005; Kachergus *et al.*, 2005; Ozelius *et al.*, 2006; Lesage *et al.*, 2010; Zabetian *et al.*, 2006*a*). Haplotype 2 has been reported in North American families of European origin (Zabetian *et al.*, 2006*a*) and French families (Lesage *et al.*, 2010). The third haplotype has been found in Japanese patients (Zabetian *et al.*, 2006*b*). We show the presence of both haplotype 1 and haplotype 2 in our patients. These distinct haplotypes suggest there have been at least two independent founding events from which the G2019S mutation arose, one likely from an ancient Middle Eastern founder (Ozelius *et al.*, 2006; Zabetian *et al.*, 2006*a*; Lesage *et al.*, 2010).

The R1441C mutation in *LRRK2* has also been found on at least two distinct haplotypes. The first haplotype is reported in a North American family originating from England (Wszolek *et al.*, 1995; Zimprich *et al.*, 2004) and in Flemish-Belgian families (Haugarvoll *et al.*, 2008; Nuytemans *et al.*, 2008), suggesting a common founder. The second haplotype is present in Italian, German, Spanish, North American and Iranian patients (Zimprich *et al.*, 2004; Haugarvoll *et al.*, 2008; Shojaee *et al.*, 2009). These haplotypes suggest that the R1441C mutation also arose in two independent events/founders, rather than a single ancient founder. Our constructed R1441C haplotypes were consistent with previous reports but we were unable to distinguish between the two different haplotypes.

We did not find any differences in motor or non-motor features between *LRRK2* carriers and non-carriers. Several studies and reviews suggest that *LRRK2* mutations are associated with a more benign disease course, less severe clinical symptoms (Nichols *et al.*, 2005), lower risk of cognitive impairment and better cognitive performance (Healy *et al.*, 2008; Srivatsal *et al.*, 2015; Kasten *et al.*, 2017). The MDSGene systematic review also suggested that *LRRK2* carriers have a good response to l-DOPA, late age at onset and absence of atypical signs (Trinh *et al.*, 2018). However, other studies have not confirmed these findings (Lesage *et al.*, 2005; Haugarvoll *et al.*, 2008; Healy *et al.*, 2008; Alcalay *et al.*, 2010*b*; Belarbi *et al.*, 2010; Ben Sassi *et al.*, 2012; Puschmann, 2013; De Rosa *et al.*, 2014; Estanga *et al.*, 2014).

*SNCA *mutations were first identified in large Parkinson’s disease families with an autosomal dominant pattern of inheritance (Polymeropoulos *et al.*, 1997; Muenter *et al.*, 1998; Singleton *et al.*, 2003). *SNCA* mutations are rare in studies of Caucasian patients (Scott *et al.*, 1999; Berg *et al.*, 2005; Nuytemans *et al.*, 2009). We found one patient carrying a heterozygous duplication, comprising 1.5% of patients reporting two or more additional family members affected by Parkinson’s disease. This is in line with previous studies reporting a mutation prevalence of 1.7% to 5.8% in familial Parkinson’s disease patients (Farrer *et al.*, 2004; Ibáñez *et al.*, 2004; Nishioka *et al.*, 2009; Bozi *et al.*, 2014).

It has been reported previously that *SNCA* mutation carriers have more frequent and more severe dementia, rapid progression, hallucinations and autonomic dysfunction (Muenter *et al.*, 1998; Farrer *et al.*, 2004; Fuchs *et al.*, 2007; Ahn *et al.*, 2008; Nishioka *et al.*, 2009; Puschmann, 2013; Bonifati, 2014; Kasten *et al.*, 2017; Schneider and Alcalay, 2017). *SNCA* triplications cause a more severe phenotype while duplications tend to cause more ‘typical’ Parkinson’s disease (Chartier-Harlin *et al.*, 2004; Ibáñez *et al.*, 2004; Hernandez *et al.*, 2016). We were not able to compare clinical features in our cohort because of the rarity of *SNCA* mutations.

Our cohort represents the largest UK-based series of *LRRK2* and *SNCA* carriers and non-carriers identified from the same unselected population, including both early and late-onset patients. In line with many previous studies, our findings suggest that Parkinson’s disease caused by *LRRK2* mutations duplications is clinically indistinguishable from sporadic disease.

### Young-onset Parkinson’s disease

We found pathogenic mutations in 6.3% (19/302) of young-onset patients, including mutations in both dominant and recessive genes. These are comparable to the frequencies previously reported in other young-onset cohorts (Alcalay *et al.*, 2010*a*; Kilarski *et al.*, 2012; Kim and Alcalay, 2017). In accordance with previous studies (Alcalay *et al.*, 2010*a*; Marder *et al.*, 2010), we show that mutations were more common in patients with earlier onset.

We identified compound heterozygous *PRKN* mutations in 2.6% of young-onset patients. While this is lower than other prevalence estimates in Caucasian populations (Abbas *et al.*, 1999; Lücking *et al.*, 2000; Lohmann *et al.*, 2003; Periquet *et al.*, 2003), our findings are in accordance with a previous UK community-based study that found that *PRKN* mutations accounted for 3.7% of patients with onset under 45 years (Kilarski *et al.*, 2012).

We also identified that 3% of patients carried single heterozygous pathogenic mutations in *PRKN* and *PINK1.* Our frequency of single heterozygous carriers is similar to what has been reported in other studies, although these include varying methods for identifying copy number variants (Klein *et al.*, 2007; Marder *et al.*, 2010).

Previous studies suggest that *PRKN* mutations are more common in familial patients (Alcalay *et al.*, 2010*a*). We found a trend for *PRKN* mutations to be more common in familial (4.2%) than in sporadic patients (1.9%), although this was not significantly different. However, 20% of patients with two additional family members affected carried *PRKN* mutations.

We found evidence for a shared haplotype for the P113Xfs mutation in five carriers across three markers spanning 242 kb. Our analysis does not include genotyping of microsatellite markers, which are needed for more detailed haplotype analysis. However, our findings are consistent with previous evidence showing that point mutations have shared haplotypes and may originate from a common founder (Farrer *et al.*, 2001; Periquet *et al.*, 2001).

*PINK1* mutation carriers were present in 0.7% of young-onset patients. This is comparable to the rate reported in a previous community-based study (Kilarski *et al.*, 2012). Mutations are more common in Asian and Italian patients (Hatano *et al.*, 2004; Valente *et al.*, 2004; Bonifati *et al.*, 2005; Li *et al.*, 2005; Tan *et al.*, 2006), reflecting population-specific allele frequencies. Our findings are consistent with the low prevalence estimates in Northern Europe and North American patients (Healy *et al.*, 2004; Rogaeva *et al.*, 2004). However, contrary to previous reports (Kilarski *et al.*, 2012), we did not find that mutations were more frequent in patients with a family history of Parkinson’s disease (1.1%) compared to sporadic patients (0.5%). This may be due to the small number of *PINK1* carriers in our cohort.

After controlling for age and disease duration, we found that *PRKN* and *PINK1* carriers had earlier age at onset, reported more postural symptoms at diagnosis and had better cognition compared to other young-onset patients. This is consistent with previous studies showing that *PRKN *and *PINK1* mutations are generally associated with slower disease progression and less cognitive impairment (Valente *et al.*, 2001, 2004; Lohmann *et al.*, 2003, 2012; Bonifati *et al.*, 2005; Tan *et al.*, 2006; Alcalay *et al.*, 2014; Bonifati, 2014; Kasten *et al.*, 2017; Kim and Alcalay, 2017). Some studies have suggested that atypical features, such as dystonia, and psychiatric symptoms may be more common in *PINK1* and *PRKN *carriers (Bonifati *et al.*, 2005; Kasten *et al.*, 2017; Koros *et al.*, 2017); however, we did not find evidence to support this. There is also substantial variability of the frequency of these symptoms in previous reports (Kasten *et al.*, 2017). Our findings are in line with a recent MDSGene systematic review, which suggested that recessive gene mutation carriers have less common cognitive decline, good treatment response and otherwise clinically typical disease (Kasten *et al.*, 2018). While a few conflicting reports suggest there are no clinical differences between *PRKN* carriers and non-carriers (Lohmann *et al.*, 2009), our findings in a large population-based study definitively show that there are clinical differences between mutation carriers and non-carriers. This may be associated with the lack of Lewy body pathology in the brain at post-mortem (Takahashi *et al.*, 1994; Mori *et al.*, 1998), although there are small numbers of *PRKN* cases with pathological data, and there is variability in findings (Farrer *et al.*, 2001; Schneider and Alcalay, 2017).

### Limitations

Our cohort was predominantly Caucasian and no pathogenic mutations were identified in non-Caucasian groups. Therefore, the estimated rate of mutations has limited application in other populations. Further large-scale studies are needed to establish mutation prevalence in other ethnic groups. Our results are also limited by the lack of complete screening; exome sequencing, MLPA and *PRKN *and *PINK1 *sequencing of all patients was not feasible due to cost limitations and the size of the cohort. Recessive gene mutations are rare in patients with older onset (Alcalay *et al.*, 2010*a*; Kilarski *et al.*, 2012); however, *PRKN* mutations have been identified in late-onset patients with onset up to 78 years (Foroud *et al.*, 2003; Klein *et al.*, 2003). Therefore, there may have been a small number of mutation carriers that were not detected with our screening methods. Our data therefore represents a minimal estimate of the frequency of genetic mutations and true numbers may be slightly higher. Our genetic prevalence rates are based on both incident and prevalent cases. We have assumed that survival and hence prevalence is not influenced by these genes, but if some genes e.g. *PRKN* and *PINK1 *are associated with better survival, then we may have under-estimated the number of cases in the general population.

A further limitation is that, while this is a large cohort study, the rarity of pathogenic mutations means that our group difference comparisons may be under-powered to detect modest phenotypic differences. Finally, our cohort is likely to still have some biases in it, given we did not undertake a rigorous community based study collecting all cases of the condition.

## Conclusions

We show that Mendelian gene mutations are a rare but significant cause of Parkinson’s disease. Patients with *PRKN* and *PINK1* mutations differ from other young-onset patients in cognition and postural symptoms. In combination with estimates of *GBA* mutation prevalence, this large-scale, relatively unbiased study suggests that up to 10% of Parkinson’s disease patients carry known genetic variants that could be targeted by new drug therapies in clinical trials and future treatment.

## Funding

The Tracking Parkinson’s study was funded by Parkinson’s UK (J-1101). M.T. is funded by a studentship from Parkinson’s UK (H-1703). Genetic analysis was funded by grants from the Medical Research Council (G1100643 to H.R.M.) and Parkinson s UK (G-1107 to N.W.W. and H.R.M.). This work was supported by the Department of Health's National Institute for Health Research Biomedical Research Centres funding.

## Competing interests

M.T. is funded by a studentship from Parkinson’s UK. Y.B-S. has received grant funding from the MRC, NIHR, Parkinson’s UK, NIH and ESRC. N.B. has received payment for advisory board attendance from UCB, Teva Lundbeck, Britannia, GSK, Boehringer and honoraria from UCB Pharma, GE Healthcare, Lily Pharma, Medtronic. He has received research grant support from GE Healthcare, Wellcome Trust, Medical Research Council, Parkinson’s UK and National Institute for Health Research. R.A.B. has received grants from Parkinson’s UK, NIHR, Cure Parkinson’s Trust, Evelyn Trust, Rosetrees Trust, MRC and EU along with payment for advisory board attendance from Oxford Biomedica LCT and FCDI and honoraria from Wiley and Springer. R.A.B. is also an NIHR Senior Investigator. D.J.B. has received grants from NIHR, Wellcome Trust, GlaxoSmithKline Ltd, Parkinson’s UK and Michael J Fox Foundation. J.H. has received honoraria from Eisai and grant support from MRC/Wellcome, Parkinson’s UK and the Michael J Fox Foundation. D.G.G. has received grants from Michael’s Movers, The Neurosciences Foundation, and Parkinson’s UK, and honoraria from UCB Pharma and GE Healthcare, and consultancy fees from Acorda Therapeutics. H.R.M. has received grants from Medical Research Council UK, Wellcome Trust, Parkinson’s UK, Ipsen Fund, Motor Neurone Disease Association, Welsh Assembly Government, PSP Association, CBD Solutions and Drake Foundation, and payment for advisory board attendance and lectures from Teva, AbbVie, Boehringer Ingelheim, and GSK. N.W.W. is supported by the MRC and NIHR UCLH Biomedical research centre. T.F. has received grants from Michael J Fox Foundation, Cure Parkinson’s Trust, Brain Research trust, John Black Charitable Foundation, Rosetrees Trust and honoraria for speaking at meetings from Bial, Profile Pharma and Medtronic.

## Supplementary material

Supplementary material is available at *Brain* online.

## Supplementary Material

awz191_Supplementary_Data

## Abbreviations

LEDD: levodopa equivalent daily dose
MLPA: multiplex ligation-dependent probe amplification
