# Supplementary material for: Genetic analysis of Mendelian mutations in a large UK population-based Parkinson’s disease study
Source: Brain. 2019 Jul 19;142(9):2828–44. doi: 10.1093/brain/awz191 (PMC6735928; doi:10.1093/brain/awz191)
Supplement: awz191_Supplementary_Data [file awz191_supplementary_data.pdf]

Genetic analysis of Mendelian mutations in a large UK population-based Parkinson's disease study.

SUPPLEMENTARY MATERIAL

Manuela M.X. Tan, Naveed Malek, Michael A. Lawton, Leon Hubbard, Alan M. Pittman, Theresita Joseph, Jason Hehir, Diane M.A. Swallow, Katherine A. Grosset, Sarah L. Marrinan, Nin Bajaj, Roger A. Barker, David J. Burn, Catherine Bresner, Thomas Foltynie, John Hardy, Nicholas Wood, Yoav Ben-Shlomo, Donald G. Grosset, Nigel M. Williams and Huw R. Morris on behalf of the PRoBaND clinical consortium

**Section**	**Description**	**Pages**
**Supplementary Text**		**2-6**
	Supplementary Methods	2-5
	Results of haplotype analysis	6
**Supplementary Tables**		**7-19**
	Supplementary Table 1: List of patients carrying pathogenic mutations, with number of additional family members affected and age at onset bracket	7-8
	Supplementary Table 2: Unique mutations identified in each gene and method by which mutation was identified	9-10
	Supplementary Table 3: Patients carrying single heterozygous mutations in recessive genes, *PRKN* and *PINK1*	11
	Supplementary Table 4: Number of cases carrying variants of unknown significance or unconfirmed pathogenicity for Parkinson's disease.	12-19
**Supplementary Figures**		**20-27**
	Supplementary Figure 1. Flow diagram of participants that were recruited and genotyped with KASP genotyping and SNP array	20
	Supplementary Figure 2. Flow diagram of genotyping for young-onset Parkinson's disease patients	21
	Supplementary Figure 3. Flow diagram of genotyping for *LRRK2* G2019S	22
	Supplementary Figure 4. Flow diagram of genotyping for *SNCA* in late-onset familial patients	23
	Supplementary Figure 5. Genotyping for *LRRK2* R1441C and other gens of interest	24
	Supplementary Figure 6. Reconstructed *LRRK2* G2019S haplotypes from imputed SNP array data	25
	Supplementary Figure 7. Reconstructed *LRRK2* R1441C haplotypes from imputed SNP array data	26
	Supplementary Figure 8. Reconstructed *PRKN* P113Xfs haplotypes from imputed SNP array data	27
**References**		**28-33**

Supplementary Methods

Research ethics

Ethics approval was provided by West of Scotland Research Ethics Service (reference 11/AL/0163). The study was carried out in accordance with the Declaration of Helsinki and is registered as NCT02881099 at ClinicalTrials.gov.

Clinical data

Family history was obtained through a standardised structured interview based on patient report. Participants were classified as having a family history if they had one or more first or second degree family members affected by Parkinson's disease.

Patients who had a family history of Parkinson's disease, with only family members affected from the same generation (siblings) were classified to be consistent with autosomal recessive inheritance. Patients with affected family members in other generations (*e.g.* father, mother, child, grandparent, aunt or uncle), were classified to be consistent with autosomal dominant inheritance. Participants that did not have a family history of Parkinson's disease were not included in either the recessive or dominant inheritance groups, although recessive disease can become manifest in apparently sporadic cases.

The criteria and calculations to determine levodopa equivalent daily dose (LEDD) dose, motor subtypes, cognitive impairment, depression and anxiety have been previously described (Malek *et al.*, 2015).

Missing AAO data were estimated from the age of diagnosis, using the mean time from disease onset to diagnosis (1.86 years) from patients with non-missing data.

Genetic analysis of mutations

Pathogenicity of a variant was classified based on MDSGene criteria: co-segregation with disease, allele frequency in healthy controls in Genome Aggregation Database (gnomAD; http://gnomad.broadinstitute.org/), the Combined Annotation Dependent Depletion (CADD) score, and functional evidence from in-vivo and in-vitro studies (Lill *et al.*, 2016; Kasten *et al.*, 2018).

Exome sequencing

Exome sequencing was performed by Macrogen (http://www.macrogen.com/) using the Agilent SureSelect capture kit (Santa Clara, CA, USA). Variant calls and individual genotypes that did not meet quality filters were excluded. Samples were aligned to the human genome (build hg19). The Genome Analysis Toolkit (GATK) was used for local realignment, base quality score recalibration, and multi-sample variant calling (Unified Genotyper). GATK Variant Quality Score Recalibration and recommended GATK training sets were used to create a high-quality set of variant calls (Farlow *et al.*, 2016). ANNOVAR was used to annotate variants with information on functional consequence, minor allele frequency (MAF), variant type and previous reporting (Wang *et al.*, 2010).

SNP array genotyping and imputation

Genotypes were recoded in Variant Call Format using PLINK1.9 (Purcell *et al.*, 2007; Chang *et al.*, 2015) and sorted using VCFtools (Danecek *et al.*, 2011). Genotypes were uploaded to the Michigan Imputation Server (https://imputationserver.sph.umich.edu/index.html) (Das *et al.*, 2016). Pre-imputation haplotype phasing was performed using Eagle and imputation was performed using Minimac3 (Das *et al.*, 2016).

Identity-by-Descent analysis

IBD analysis was conducted in PLINK v.1.9 using linkage-pruned SNPs with MAF>0.05. SNPs were excluded if they had a squared correlation (r^2) of greater than 0.05 within a sliding window of 50 adjacent SNPs scrolling through 5 SNPs at a time. IBD analysis was conducted using default PLINK settings which assesses extended segmental sharing of at least 100 SNPs and total length greater than 1 megabase (Mb) (Purcell *et al.*, 2007).

Haplotypes

For *LRRK2* G2019S haplotypes, the following markers were used to construct haplotypes: rs10878245, rs28903073, rs7966550, rs1896252, rs1427263, rs11176013, rs11564148, rs2404834, rs34637584, rs10784522, rs10878405, rs33962975 (ss52051244) and rs3761863 (Supplementary Figure 6) (Zabetian *et al.*, 2006b, a). All markers were directly genotyped or imputed with very high accuracy ($r^2 \geq 0.98$) except for rs28903073 which had an imputation quality

score (r^2) of 0.56. Haplotypes 1 and 2 were defined according to Zabetian and colleagues (Zabetian *et al.*, 2006b, a) in which 5 intragenic SNPs rs28903073, rs2404834, rs10784522, rs10878405 and rs33962975 distinguish the two haplotypes. Haplotype 1 is the most common and has been reported in in European, Ashkenazi Jewish and Arab (Lesage *et al.*, 2010) individuals, while Haplotype 2 has been found in a few non-Jewish/north African European families (Zabetian *et al.*, 2006a).

For the *LRRK2* R1441C mutation, the following markers spanning approximately 127kB were used to construct haplotypes: rs10878245, rs10878246, rs41286474, rs1896252, rs1427263, rs11176013, rs11564148, rs11564205, rs10878405, and rs3761863 (Nuytemans *et al.*, 2008). This is shown in Supplementary Figure 7.

For the *PRKN* P113X frameshift (P113Xfs) mutation, the following markers spanning approximately 1Mb were used to construct haplotypes: rs1801334, rs1801582, rs3765474, rs1801474, rs4709583, and rs2075923 (Periquet *et al.*, 2001; Oliveira *et al.*, 2003), shown in Supplementary Figure 8.

Mutations of uncertain pathogenicity

We reported variants that were suggested to be potentially pathogenic or protective for Parkinson's disease/parkinsonism from previous studies (excluding those reported in the main text), with references provided in Supplementary Table 3. We screened all the *PARK* loci (Hernandez *et al.*, 2016) and genes associated with parkinsonism in MDSGene (Lill *et al.*, 2016). Additional genes and variants were identified through ClinVar (https://www.ncbi.nlm.nih.gov/clinvar/), Genomics England PanelApp Parkinson's disease panel (https://panelapp.genomicsengland.co.uk/), Online Mendelian Inheritance in Man (https://www.omim.org/) and from literature searches.

APOE ε4 genotypes were extracted from exome data using SNPs rs429358 and rs7412 (Williams-Gray *et al.*, 2009).

Literature review

For our discussion of previous literature, we focused on studies relating to the frequency and phenotype-genotype of *LRRK2*, *SNCA*, *PRKN* and/or *PINK1* pathogenic mutations. We did not conduct a systematic review of the literature but used MDSGene systematic reviews (Kasten *et*

al., 2018; Trinh *et al.*, 2018) and other recent review articles (from 2013 onwards) to identify relevant studies and present a balanced discussion of previous literature.

Results of haplotype analysis

Haplotype construction showed that 6 of 16 *LRRK2* G2019S patients carried Haplotype 2 (European predominant), and 9 carried Haplotype 1 (Jewish/European predominant). Haplotype could not be confirmed for one patient, possibly due to lower imputation quality for one marker rs28903073 (Supplementary Figure 6).

Haplotype construction for the two *LRRK2* R1441C carriers showed they shared a common haplotype based on region of 126 kB across 10 SNP markers, indicating that these apparently unrelated individuals shared a common ancestral founder (Supplementary Figure 7). This shared region is consistent with haplotypes previously reported in European patients (Haugarvoll *et al.*, 2008; Criscuolo *et al.*, 2011). However we were unable to distinguish between the two common haplotypes, the Belgian-Western Nebraska haplotype (Nuytemans *et al.*, 2008) or the other major haplotype found in Italian, German, Spanish and American patients.

The most common *PRKN* mutations detected were P113Xfs deletion (5/8 carriers) and the R275W mutation (4/8 carriers). Haplotype reconstruction suggests that the five P113Xfs mutation carriers had a shared segment spanning at least 242 kB, at the markers rs1801474, rs4709583 and rs2075923, indicating a common founder (Supplementary Figure 8). Haplotypes were discordant at the markers rs1801582 and rs3765474.

For *PRKN* R275W, there were 3 carriers with SNP array data and haplotypes appeared to be shared at all 6 markers genotyped spanning approximately 1Mb (data not shown). One *PRKN* carrier did not have SNP array data available for haplotype reconstruction. Only two patients carried deletions but these were in different exons so we were unable to observe shared or discordant haplotypes.

Supplementary Tables

Supplementary Table 1. Patients carrying pathogenic mutations. Wild type (WT) genotypes are indicated as well as any additional mutations carried. For each patient, the number of other family members affected by Parkinson's disease is indicated.

ID	Gene	Allele 1	Allele 2	Additional mutations	Number of other family members affected by Parkinson's disease	Age at onset bracket
1	*LRRK2*	G2019S	WT	*GBA* p.E326K	0	40-49
2	*LRRK2*	G2019S	WT	*GBA* p.E326K	0	40-49
3	*LRRK2*	G2019S	WT	*GBA* p.E326K/ p.P122H	1	40-49
4	*LRRK2*	G2019S	WT		0	30-39
5	*LRRK2*	G2019S	WT		1	60-69
6	*LRRK2*	G2019S	WT		0	60-69
7	*LRRK2*	G2019S	WT		1	60-69
8	*LRRK2*	G2019S	WT		3	30-39
9	*LRRK2*	G2019S	WT		0	60-69
10	*LRRK2*	G2019S	WT		1	40-49
11	*LRRK2*	G2019S	WT		0	70-79
12	*LRRK2*	G2019S	WT		1	40-49
13	*LRRK2*	G2019S	WT		0	60-69
14	*LRRK2*	G2019S	WT		1	40-49
15	*LRRK2*	G2019S	WT		1	60-69
16	*LRRK2*	G2019S	WT		0	40-49
17	*LRRK2*	R1441C	WT		3	60-69

18	*LRRK2*	R1441C	WT		2	50-59
19	*PINK1*	Exon 1 deletion	W90Xfs		1	40-49
20	*PINK1*	Exon 5 deletion	Exon 5 deletion		0	30-39
21	*PRKN*	P113Xfs	R275W	*PRKN* R33X	1	≤ 20
22	*PRKN*	P113Xfs	G430D		2	30-39
23	*PRKN*	P113Xfs	R275W		0	30-39
24	*PRKN*	Exon 1 deletion	R275W		0	40-49
25	*PRKN*	Exon 4 deletion	R275W		0	≤ 20
26	*PRKN*	Q34Xfs	P113Xfs		0	40-49
27	*PRKN*	R33X	G430D		2	30-39
28	*PRKN*	Exon 5 deletion	P113Xfs		2	20-29
29	*SNCA*	Exon 1-6 duplication	WT		2	50-59

Supplementary Table 2. Unique mutations identified in each gene, including the protein level change, cDNA change, reference transcript, and method by which the mutation was identified. If a mutation was identified with more than one method (e.g. identified through KASP assay and confirmed through exome sequencing), both methods are listed.

**Gene**	**Protein level**	**cDNA level**	**Reference transcript ID (GRCh37)**	**Method identified**
LRRK2	p.G2019S	c.6055G>A	NM_198578	KASP assay<br>Exome sequencing
LRRK2	p.R1441C	c.4321C>T	NM_198578	Exome sequencing
SNCA	Exon 1-6 duplication		NM_000345.3	MLPA
PRKN	p.P113Xfs	c.337_376del40	NM_004562/<br>NM_013987	Sanger sequencing<br>Exome sequencing
PRKN	p.R275W	c.823C>T	NM_004562	Sanger sequencing<br>Exome sequencing
PRKN	p.G430D	c.1289G>A	NM_004562	Sanger sequencing<br>Exome sequencing
PRKN	p.R33X	c.97C>T	NM_013988/<br>NM_013987/<br>NM_004562	Sanger sequencing<br>Exome sequencing
PRKN	p.Q34Xfs	c.101_102delAG	NM_004562/<br>NM_013987/<br>NM_013988	Sanger sequencing<br>Exome sequencing
PRKN	Exon 1 deletion		NM_00456.2	MLPA
PRKN	Exon 4 deletion		NM_00456.2	MLPA
PRKN	Exon 5 deletion		NM_00456.2	MLPA
PINK1	p.W90Xfs	c.270G>A	NM_032409	Sanger sequencing
PINK1	Exon 1 deletion		NM_032409.2	MLPA
PINK1	Exon 5 deletion		NM_032409.2	MLPA

MLPA = Multiplex Ligation-dependent Probe Amplification

KASP = ‘Kompetitive’ allele-specific polymerase chain reaction assay.

Supplementary Table 3. Patients carrying single heterozygous mutations in recessive genes, *PRKN* and *PINK1*. Patients carrying variants that did not meet the MDSGene criteria for pathogenicity are not reported.

**ID**	**Gene**	**Protein level**	**cDNA level**
30	*PRKN*	Exon 8 deletion	
31	*PRKN*	Exon 8 duplication	
32	*PRKN*	Exon 2 deletion	
33	*PRKN*	Exon 5-6 deletion	
34	*PRKN*	Q34Xfs	c.101delA
35	*PRKN*	R275W	c.823C>T
36	*PINK1*	Exon 5 deletion	
37	*PINK1*	Exon 1 duplication	
38	*PINK1*	R279H	c.836G>A

Supplementary Table 4. Number of cases carrying variants of unknown significance or unconfirmed pathogenicity for Parkinson’s disease. Allele frequency in our cohort (as a proportion of 489 patients that were exome sequenced) is compared with allele frequency in control populations (all populations) from gnomAD unless otherwise specified.

**Locus**	**Gene**	**Inheritance pattern**	**Mutation**	**Number of carriers**	**Allele frequency**	**Control allele frequency (gnomAD)**	**Previously reported in Parkinson’s disease (PD)**
***PARK* loci**							
Park5	*UCHL1*	Autosomal dominant	p.S18Y	274 heterozygous<br>53 homozygous	0.389	0.2282	Suggested to be protective against PD but lack of replication (Wintermeyer *et al.*, 2000; Maraganore *et al.*, 2004; Healy *et al.*, 2006)
Park9	*ATP13A2*	Autosomal recessive	p.G504R	No carriers	0	Not reported	Recessive inheritance for Kufkor-Rakeb syndrome (juvenile onset parkinsonism).(Fonzo *et al.*, 2013)
Park11	*GIGYF2*	Autosomal dominant	p.1209_1212del	2 heterozygous	0.002	0.000 (Lautier *et al.*, 2008)	Small deletion found in both cases and controls, likely not associated with increased PD risk (Lautier *et al.*, 2008; Bras *et al.*, 2009; Nichols *et al.*, 2009)
Park13	*HTRA2*	Autosomal dominant	p.G399S	3 heterozygous	0.003	0.004	Mixed evidence for association with increased risk of PD
			p.A141S	19 heterozygous	0.019	0.019	
			p.L72P	3 heterozygous	0.003	0.002	

							(Strauss *et al.*, 2005; Bogaerts *et al.*, 2008; Simón-Sánchez and Singleton, 2008; Unal Gulsuner *et al.*, 2014)
Park14	*PLA2G6*	Autosomal recessive	p.R741Q	No carriers	0	0.0000876	Homozygous mutations found in dystonia-parkinsonism (Paisan-Ruiz *et al.*, 2009)
			p.R747W	No carriers	0	0.00002693	
Park15	*FBXO7*	Autosomal recessive	p.R498X	No carriers	0	0.000004062	Homozygous and compound heterozygous mutations found in early onset parkinsonism pallido-pyramidal syndrome (Shojaee *et al.*, 2008; Di Fonzo *et al.*, 2009)
			p.T22M	No carriers	0	Not reported	
			p.R378G	No carriers	0	0.00004691	
Park18	*EIF4G1*	Autosomal dominant	p.R1205H	No carriers	0	0.0002069	Previously reported in familial and sporadic PD (Chartier-Harlin *et al.*, 2011) but not confirmed in later studies (Nichols *et al.*, 2015)
			p.A502V	No carriers	0	0.00006928	
			p.R1197W	No carriers	0	0.00004712	
			p.G686C	No carriers	0	0.0001407	
			p.S1164R	No carriers	0	0.00001219	
Park19	*DNAJC6*	Autosomal recessive	p.R927G<br>p.T741T	No carriers	0	Not reported	Homozygous mutations found in large family with Parkinson’s disease (Olgiati *et al.*, 2016)

			p.Q734X	No carriers	0	0.000004063	Homozygous mutations found in large consanguineous Turkish family with Parkinson's disease and mental retardation (Köroĝlu *et al.*, 2013)
			p.Q789X	No carriers	0	Not reported	Homozygous mutations found in consanguineous Yemeni family with juvenile-onset Parkinson's disease (Elsayed *et al.*, 2016)
**DYT loci**							
DYT1	*TOR1A*	Autosomal dominant	p.D216H	122 heterozygous<br>14 homozygous	0.153	0.1236	H allele is protective against dystonia in carriers of the *TOR1A* 302GAG deletion (Risch *et al.*, 2007; Kamm *et al.*, 2008)
DYT3	*TAF1*	X-linked	rs41484056 (g.70432420A>G)<br>rs41377154 (g.70482458G>A)<br>rs41532445 (g.70559687G>A)<br>rs41438158 (g.70853421C>T)	No carriers	0	Not reported	Suggested disease-specific haplotype associated with X-linked dystonia/ parkinsonism (Domingo *et al.*, 2015)
DYT5a	*GCH1*		p.K224R	No carriers	0	0.0003641	

		Autosomal dominant	p.V402I	1 heterozygous	0.001022495	0.0002016	Identified in dopa-responsive dystonia and PD (Leuzzi *et al.*, 2002; Mencacci *et al.*, 2014)
			p.Q110X	No carriers	0	Not reported	
			p.M230I	No carriers	0	Not reported	
DYT6	*THAP1*	Autosomal dominant	p.M1?	No carriers	0	Not reported	Predominantly early-onset, generalised or segmental dystonia (Zittel *et al.*, 2010; Saunders-Pullman *et al.*, 2014; Krause *et al.*, 2015)
			p.F45Lfs73X	No carriers	0	Not reported	
			p.R13H	No carriers	0	Not reported	
DYT16	*PRKRA*	Autosomal recessive	p.P222L	1 heterozygous	0.001	0.00008	Recessive inheritance for early-onset dystonia DYT16 (Camargos *et al.*, 2008; Zech *et al.*, 2014)
DYT18	*SLC2A1*	Autosomal dominant	p.R458W	No carriers	0	Not reported	Associated with glucose transporter 1 deficiency causing generalised epilepsies (Arsov *et al.*, 2012; De Giorgis *et al.*, 2015)
**Atypical parkinsonism**							
	DCTN1	Autosomal dominant	p.G71E<br>p.G71A<br>p.G71R	No carriers	0	Not reported	Associated with Perry syndrome (Ohshima *et al.*, 2010; Chung *et al.*, 2014; Tacik *et al.*, 2014; Konno *et al.*,
			p.F52L	No carriers	0	Not reported	
			p.Y78C	No carriers	0	Not reported	

			p.G67D	No carriers	0	Not reported	2017; Umemoto *et al.*, 2017)
			p.Q74P	No carriers	0	Not reported	
			p.K56R	No carriers	0	0.00001767	Identified in patients with PSP (Gustavsson *et al.*, 2016)
	SYNJ1	Autosomal recessive	p.R258Q	No carriers	0	0.00001193	Associated with early-onset parkinsonism (Krebs *et al.*, 2013; Quadri *et al.*, 2013)
			p.R459P	No carriers	0	Not reported	
**Risk variants for PD**							
	MAPT	Risk variant	p.A544A	149 heterozygous 18 homozygous	0.189	0.1424	Conflicting evidence for association with PD risk, although meta-analysis shows that the H1/H1 is associated with increased risk of PD (Martin *et al.*, 2001; Healy *et al.*, 2004; Davis *et al.*, 2016)
	APOE	Risk variant	ε4 allele	15 homozygous 99 heterozygous	0.132	0.13 to 0.14 (Gao *et al.*, 2011; Federoff *et al.*, 2012)	Mixed evidence as to whether the ε4 allele is associated with increased risk for PD and potentially dementia in PD (Marder *et al.*, 1994; Li *et al.*, 2004; Gao *et al.*, 2011; Federoff *et al.*, 2012)
**Other genes for PD including recently reported genes**							
	CHCHD2	Autosomal dominant	p.P2L	1 heterozygous	0.001	0.001	Suggested to increase PD risk, found in

							Chinese populations but not European (Foo *et al.*, 2015; Jansen *et al.*, 2015)
	PANK2	Autosomal recessive	p.D46V	3 heterozygous	0.003	0.003443	Suggested to be associated with typical PD. No difference between allele frequencies for controls and PD cases (Klopstock *et al.*, 2005)
			p.L111Q	63 heterozygous<br>3 homozygous	0.071	0.07991	
			p.G126A	90 heterozygous<br>357 homozygous	0.822	0.8686	
	C19orf12	Autosomal recessive	p.K142E	3 heterozygous	0.003	0.00212	Compound heterozygous mutations associated with PD and neurodegeneration with brain iron accumulation presenting with parkinsonism (Hartig *et al.*, 2011)
	SNCAIP	Autosomal dominant	p.R621C	8 heterozygous	0.008	0.003	Conflicting evidence for association with PD risk (Marx *et al.*, 2003; Myhre *et al.*, 2008)
	SLC41A1	Polymorphism (additive/recessive model)	p.T113T	208 heterozygous<br>45 homozygous	0.304	0.287	Potentially associated with decreased risk of PD in Iranian and Chinese cohorts, but not in European cohorts (Tucci *et al.*,

							2010; Wang *et al.*, 2015; Madadi *et al.*, 2016)
	LRP10	Autosomal dominant	p. P699S	No carriers	0	0.00001623	Reported in familial PD, PD dementia, and dementia with Lewy bodies (Quadri *et al.*, 2018)
			p.G603R	No carriers	0	0.00002286	
			c.1424+5delG	No carriers	0	0.0000201	
			p. R533L	No carriers	0	Not reported	
			p. Y307N	No carriers	0	0.0000597	
			c. 1424+5G→A	No carriers	0	Not reported	
			p.R235C	No carriers	0	0.00002476	
			p. N517del	No carriers	0	Not reported	
			p. A212Sfs*17	No carriers	0	Not reported	
			p.A121fs Frameshift del	1 heterozygous	0.001022495	Not reported	Not previously reported
			p.Y200C	1 heterozygous	0.001022495	0.00004376	Not previously reported
			p.Q218X stopgain	1 heterozygous	0.001022495	0.00001769	Not previously reported
			p.T278K	1 heterozygous	0.001022495	Not reported	Not previously reported
			p.P354S	1 heterozygous	0.001022495	Not reported	Not previously reported
			p.R395C	1 heterozygous	0.001022495	0.00005660	Not previously reported

PD = Parkinson’s disease

Supplementary Figures

Supplementary Figure 1. Flow diagram of participants that were recruited and genotyped with KASP genotyping and SNP array.

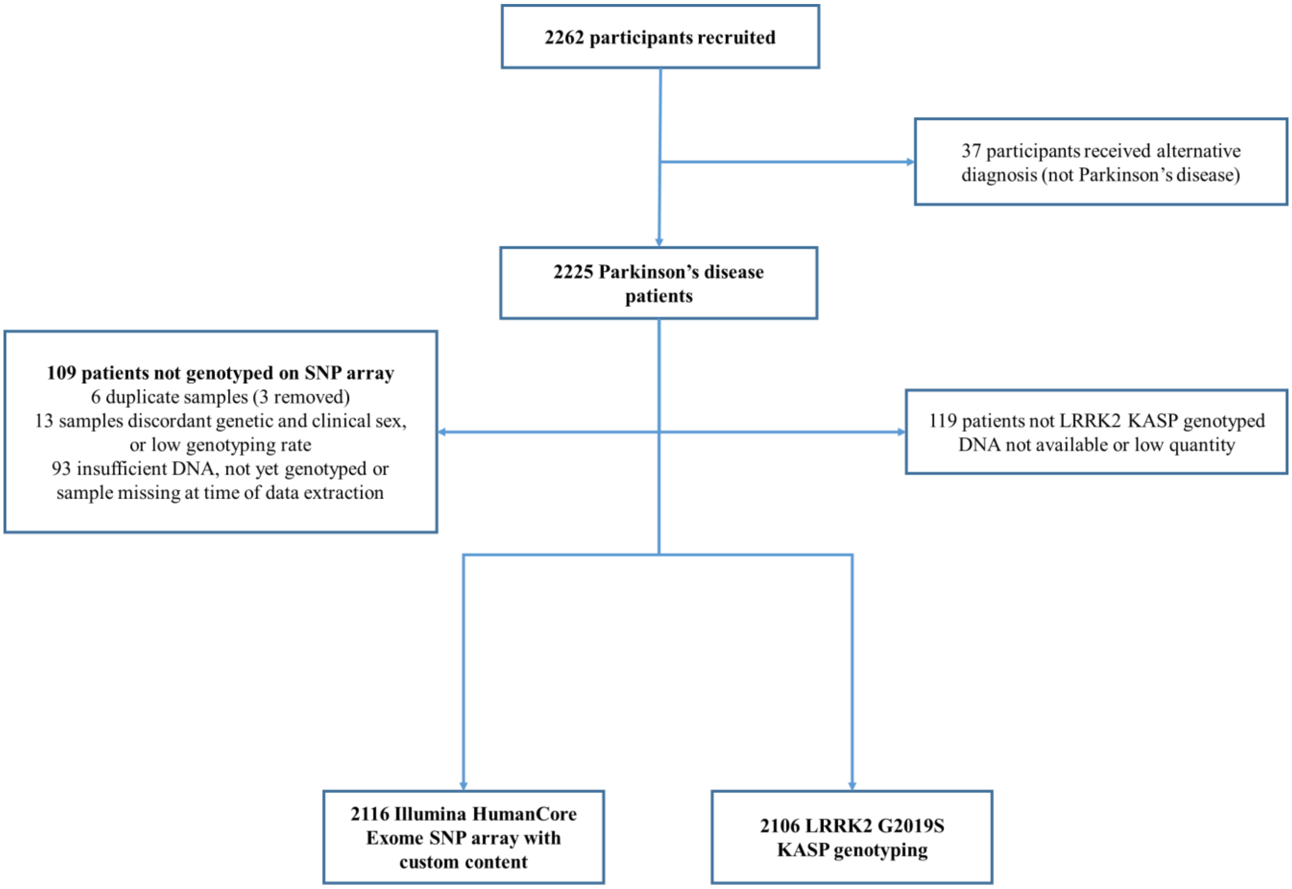

KASP = ‘Kompetitive’ allele-specific polymerase chain reaction assay for *LRRK2* G2019S

Supplementary Figure 2. Flow diagram of genotyping for young-onset Parkinson's disease patients. Grey shaded boxes indicate the samples that were included for final analysis of *PRKN* and *PINK1*.

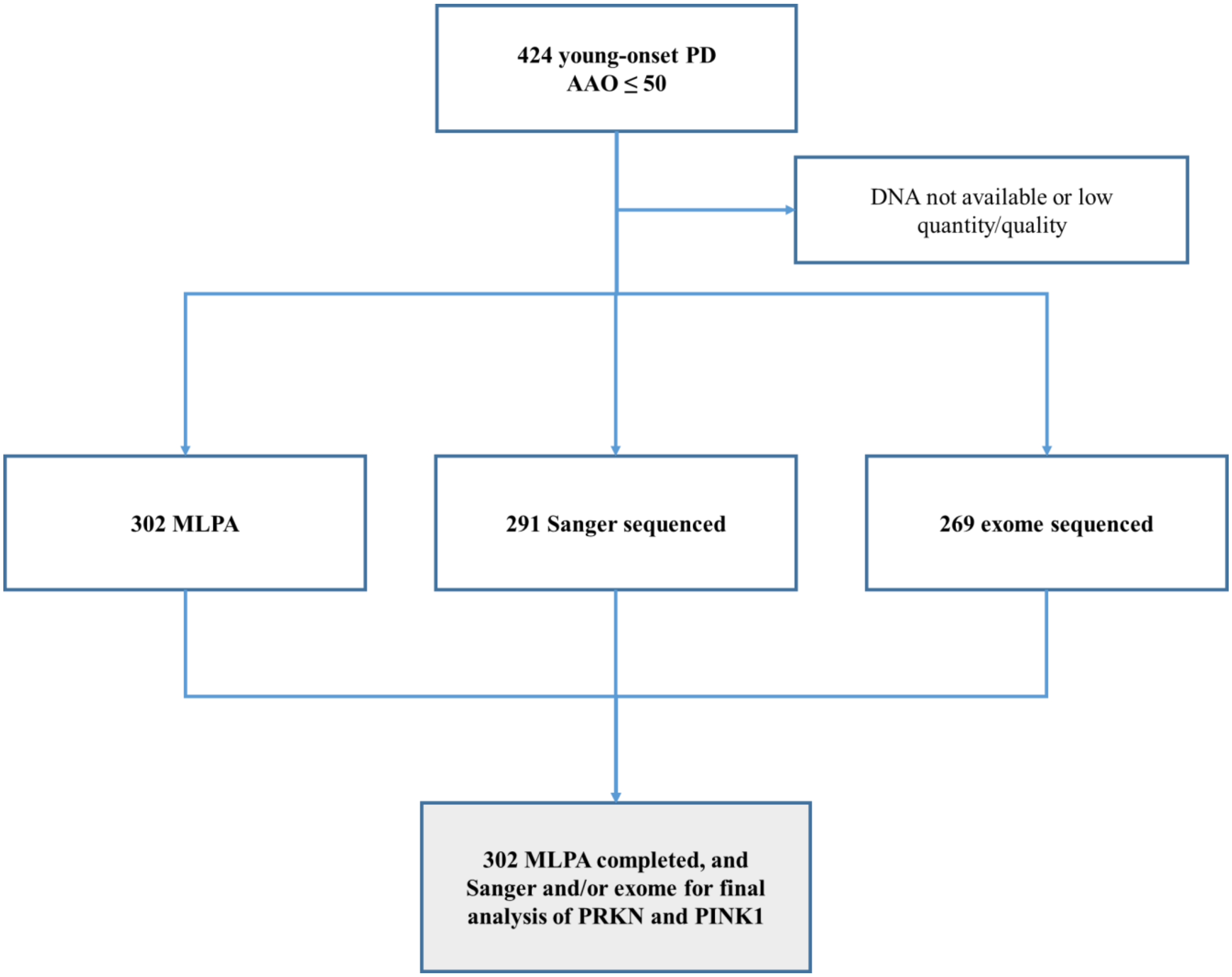

Supplementary Figure 3. Flow diagram of genotyping for *LRRK2* G2019S. Grey shaded boxes indicate the samples that were included for final analysis of *LRRK2* G2019S.

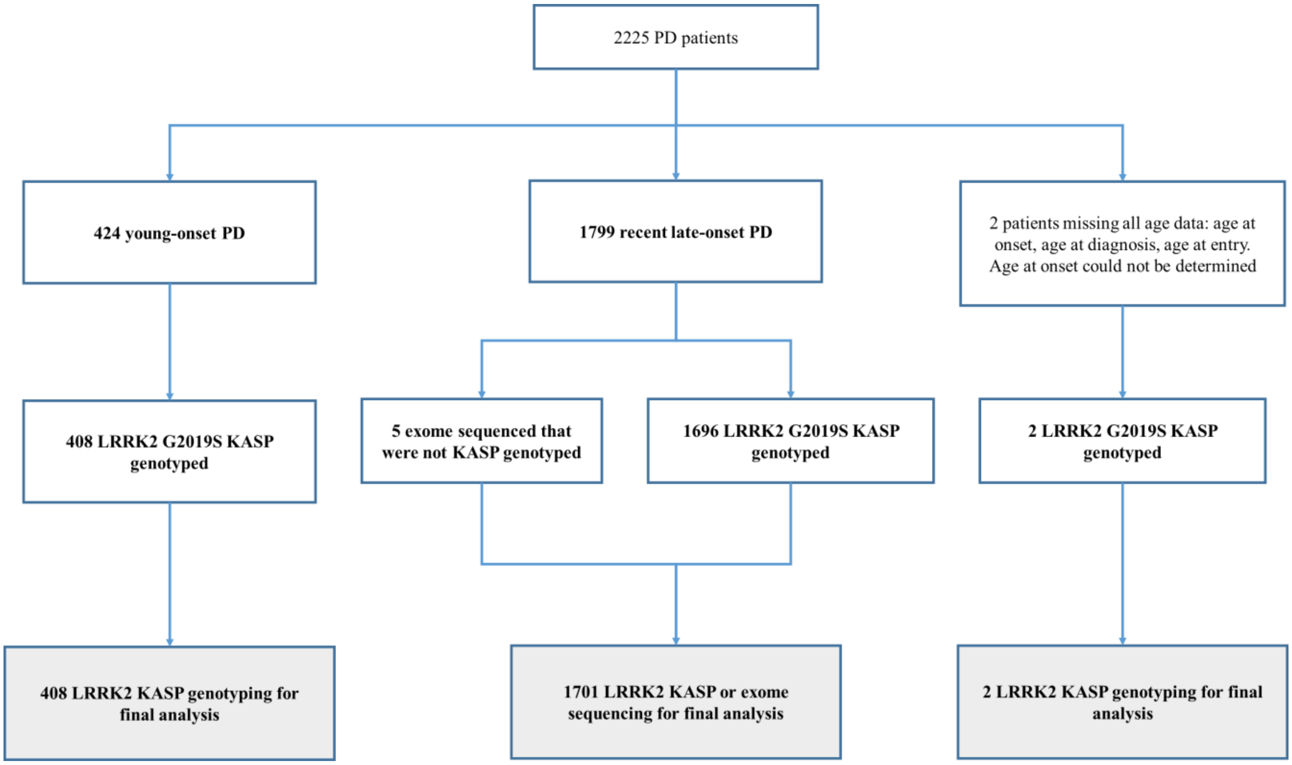

Supplementary Figure 4. Flow diagram of genotyping for *SNCA* in late-onset familial patients.

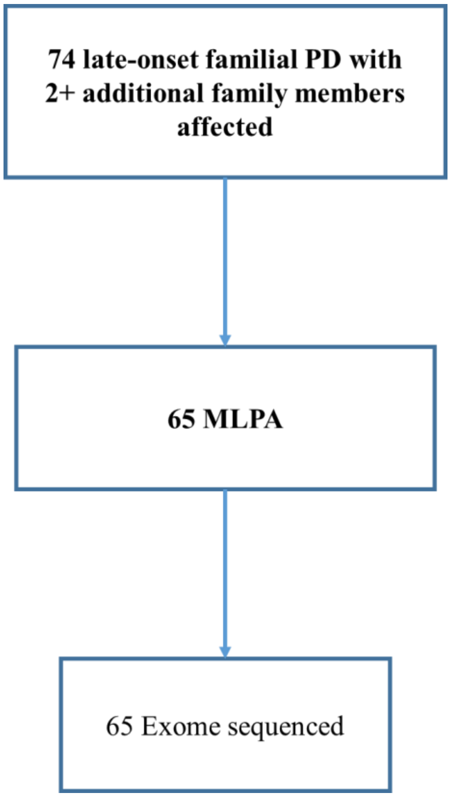

Supplementary Figure 5. Genotyping for *LRRK2* R1441C and other genes of interest.

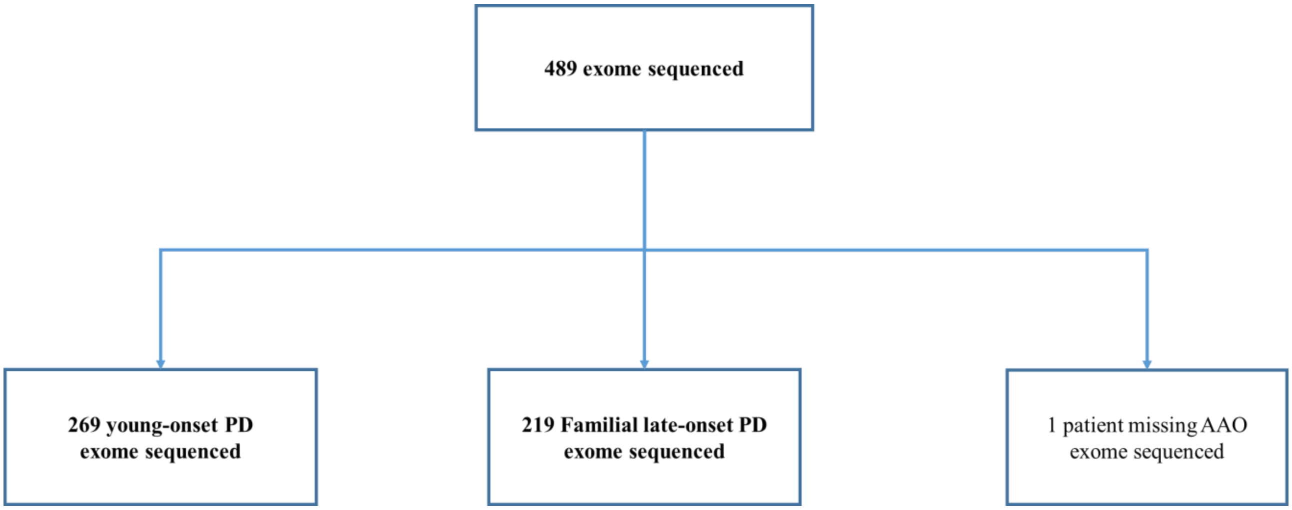

Supplementary Figure 6. Reconstructed *LRRK2* G2019S haplotypes from imputed SNP array data. For markers where phase could not be unambiguously determined, both alleles are shown in brackets. Blue shading denotes haplotype 1, orange shading denotes haplotype 2 and red shading is used to denote a haplotype that does not fit with either haplotype 1 or 2. The yellow highlight indicates the pathogenic mutation G2019S that is shared by all carriers. Markers in bold can be used to delineate the different haplotypes. All markers were imputed with very high accuracy ($r^2 \geq 0.98$) except for rs28903073. This may explain the discordant haplotype generated for Patient 12.

SNP	Position	Patient 11	Patient 8	Patient 13	Patient 6	Patient 9	Patient 1	Patient 3	Patient 4	Patient 2	Patient 10	Patient 15	Patient 7	Patient 5	Patient 16	Patient 14	Patient 12
rs10878245	12:40631791	C	C	C	(C/T)	(C/T)	C	C	C	C	C	(C/T)	C	C	(C/T)	C	C
**rs28903073**	12:40653510	**(A/G)**	**(A/G)**	**(A/G)**	**(A/G)**	**(A/G)**	**(A/G)**	**(A/G)**	**(A/G)**	**(A/G)**	**G**	**G**	**G**	**G**	**G**	**G**	**G**
rs7966550	12:40688695	T	T	T	T	(C/T)	T	T	T	T	T	T	T	T	T	T	T
rs1896252	12:40713759	C	(C/T)	C	C	(C/T)	C	C	C	C	C	C	C	(C/T)	(C/T)	(C/T)	(C/T)
rs1427263	12:40713834	A	(A/C)	A	A	(A/C)	A	A	A	A	A	A	A	(A/C)	A	(A/C)	(A/C)
rs11176013	12:40713873	G	(A/G)	G	G	(A/G)	G	G	G	G	G	G	G	(A/G)	(A/G)	(A/G)	(A/G)
rs11564148	12:40713901	A	(A/T)	(A/T)	(A/T)	(A/T)	A	A	A	A	A	A	(A/T)	(A/T)	(A/T)	(A/T)	(A/T)
**rs2404834**	12:40729007	**C**	**C**	**C**	**C**	**C**	**C**	**C**	**C**	**C**	**(C/T)**	**(C/T)**	**(C/T)**	**(C/T)**	**(C/T)**	**(C/T)**	**C**
**rs34637584**	**G2019S**	**A**	**A**	**A**	**A**	**A**	**A**	**A**	**A**	**A**	**A**	**A**	**A**	**A**	**A**	**A**	**A**
**rs10784522**	12:40740365	**T**	**(G/T)**	**(G/T)**	**(G/T)**	**(G/T)**	**T**	**T**	**T**	**T**	**(G/T)**	**(G/T)**	**(G/T)**	**G**	**G**	**G**	**(G/T)**
**rs10878405**	12:40742254	**A**	**(A/G)**	**(A/G)**	**(A/G)**	**(A/G)**	**A**	**A**	**A**	**A**	**(A/G)**	**(A/G)**	**(A/G)**	**G**	**G**	**G**	**(A/G)**
**ss52051244 (rs33962975)**	12:40757330	**A**	**A**	**A**	**A**	**A**	**A**	**A**	**A**	**A**	**(A/G)**	**(A/G)**	**(A/G)**	**(A/G)**	**(A/G)**	**A**	**A**
rs3761863	12:40758652	C	C	C	C	(C/T)	C	C	C	C	C	C	C	(C/T)	C	C	(C/T)

Supplementary Figure 7. Reconstructed *LRRK2* R1441C haplotypes from imputed SNP array data, all markers had high imputation quality ($r^2 \geq 0.98$). Markers that distinguish previously defined haplotypes are indicated in bold. The yellow highlight indicates the pathogenic mutation R1441C that is shared by all carriers.

SNP	Position	Patient 17	Patient 18
**rs10878245**	12:40631791	(C/T)	(C/T)
**rs10878246**	12:40632099	T	T
**R1441C**	**12:40704236**	**T**	**T**
rs41286474	12:40704557	T	T
**rs1896252**	12:40713759	(C/T)	(C/T)
**rs1427263**	12:40713834	(A/C)	(A/C)
**rs11176013**	12:40713873	(A/G)	(A/G)
**rs11564148**	12:40713901	(A/T)	(A/T)
rs11564205	12:40714009	A	A
**rs10878405**	12:40742254	(A/G)	(A/G)
**rs11176143**	12:40742363	(A/G)	(A/G)
**rs3761863**	12:40758652	(C/T)	(C/T)

Supplementary Figure 8. Reconstructed *PRKN* P113Xfs haplotypes from imputed SNP array data. When phase could not be determined at heterozygous markers, both alleles are shown. Shared segments are highlighted in green. The yellow highlight indicates the pathogenic mutation P113Xfs. that is shared by all carriers.

		Mutations	Patient 22<br>p.P113Xfs / G430D	Patient 26<br>p.Q34Xfs / p.P113Xfs	Patient 28<br>p.P113Xfs / PARK2 Exon5 hemizygous deletion	Patient 23<br>p.P113Xfs / p.R275W	Patient 21<br>p.P113Xfs / p.R275W / R33X
**SNP**	**Effect**	**Position**					
rs1801334	p.Asp394Asn	161781225	C	C	C	C	(C/T)
rs1801582	p.Val380Leu	161807855	G	C	(C/G)	C	C
rs3765474	IVS7-35 G>A	161990483	(C/T)	(C/T)	T	C	(C/T)
rs1801474	p.Ser167Asn	162622197	C	C	C	C	C
rs4709583	IVS3-20 T>C	162622304	G	G	G	G	G
	**p.P113Xfs**	**162683593 - 162683632**	**40bp del**	**40bp del**	**40bp del**	**40bp del**	**40bp del**
rs2075923	IVS2+25 T>C	162864317	A	A	A	A	A

References

Arsov T, Mullen SA, Rogers S, Phillips AM, Lawrence KM, Damiano JA, et al. Glucose transporter 1 deficiency in the idiopathic generalized epilepsies. Ann. Neurol. 2012; 72: 807–815.

Bogaerts V, Nuytemans K, Reumers J, Pals P, Engelborghs S, Pickut B, et al. Genetic variability in the mitochondrial serine protease HTRA2 contributes to risk for Parkinson disease. Hum. Mutat. 2008; 29: 832–840.

Bras J, Simon-Sanchez J, Federoff M, Morgadinho A, Januario C, Ribeiro M, et al. Lack of replication of association between GIGYF2 variants and Parkinson disease. Hum. Mol. Genet. 2009; 18: 341–346.

Camargos S, Scholz S, Simón-Sánchez J, Paisán-Ruiz C, Lewis P, Hernandez D, et al. DYT16, a novel young-onset dystonia-parkinsonism disorder: identification of a segregating mutation in the stress-response protein PRKRA. Lancet Neurol. 2008; 7: 207–215.

Chang CC, Chow CC, Tellier LCAM, Vattikuti S, Purcell SM, Lee JJ. Second-generation PLINK: Rising to the challenge of larger and richer datasets. Gigascience 2015; 4: 1–16.

Chartier-Harlin MC, Dachsel JC, Vilariño-Güell C, Lincoln SJ, Leprêtre F, Hulihan MM, et al. Translation initiator EIF4G1 mutations in familial parkinson disease. Am. J. Hum. Genet. 2011; 89: 398–406.

Chung EJ, Hwang JH, Lee MJ, Hong JH, Ji KH, Yoo WK, et al. Expansion of the clinicopathological and mutational spectrum of Perry syndrome. Park. Relat. Disord. 2014; 20: 388–393.

Criscuolo C, De Rosa A, Guacci A, Simons EJ, Breedveld GJ, Peluso S, et al. The LRRK2 R1441C mutation is more frequent than G2019S in Parkinson’s disease patients from Southern Italy. Mov. Disord. 2011; 26: 1732–1736.

Danecek P, Auton A, Abecasis G, Albers CA, Banks E, DePristo MA, et al. The variant call format and VCFtools. Bioinformatics 2011; 27: 2156–2158.

Das S, Forer L, Schönherr S, Sidore C, Locke AE, Kwong A, et al. Next-generation genotype imputation service and methods. Nat. Genet. 2016; 48: 1284–1287.

Davis A a., Andruska KM, Benitez B a., Racette B a., Perlmutter JS, Cruchaga C. Variants in GBA, SNCA, and MAPT Influence Parkinson Disease Risk, Age at Onset, and Progression. Neurobiol. Aging 2016; 37: 209.e1-209.e7.

Domingo A, Westenberger A, Lee L V., Brænne I, Liu T, Vater I, et al. New insights into the genetics of X-linked dystonia-parkinsonism (XDP, DYT3). Eur. J. Hum. Genet. 2015; 23: 1334–1340.

Elsayed LEO, Drouet V, Usenko T, Mohammed IN, Hamed AAA, Elseed MA, et al. A Novel Nonsense Mutation in DNAJC 6 Expands the Phenotype of Autosomal-Recessive Juvenile-Onset Parkinson’s Disease. Ann. Neurol. 2016; 79: 335–337.

Farlow JL, Robak LA, Hetrick K, Bowling K, Boerwinkle E, Coban-Akdemir ZH, et al. Whole-Exome Sequencing in Familial Parkinson Disease. JAMA Neurol. 2016; 73: 68–75.

Federoff M, Jimenez-Rolando B, Nalls MA, Singleton AB. A large study reveals no association between APOE and Parkinson's disease. Neurobiol. Dis. 2012; 46: 389–392.

Fonzo A Di, Chien HF, Socal M, Giraudo S, Tassorelli C, Iliceto G, et al. ATP13A2 missense mutations in juvenile parkinsonism and young onset Parkinson disease. Neurology 2013; 68: 1557–1562.

Di Fonzo A, Dekker MCJ, Montagna P, Baruzzi A, Yonova EH, Guedes LC, et al. FBXO7 mutations cause autosomal recessive, early-onset parkinsonian-pyramidal syndrome. Neurology 2009; 72: 240–245.

Foo JN, Liu J, Tan EK. CHCHD2 and Parkinson's disease. Lancet Neurol. 2015; 14: 681–682.

Gao J, Huang X, Park Y, Liu R, Hollenbeck A, Schatzkin A, et al. Apolipoprotein E genotypes and the risk of Parkinson disease. Neurobi 2011; 32: 2106.e1-2106.e6.

De Giorgis V, Teutonico F, Cereda C, Balottin U, Bianchi M, Giordano L, et al. Sporadic and familial glut1ds Italian patients: A wide clinical variability. Seizure 2015; 24: 28–32.

Gustavsson EK, Trinh J, Guella I, Szu-Tu C, Khinda J, Lin CH, et al. DCTN1 p.K56R in progressive supranuclear palsy. Park. Relat. Disord. 2016; 28: 56–61.

Hartig MB, Iuso A, Haack T, Kmiec T, Jurkiewicz E, Heim K, et al. Absence of an orphan mitochondrial protein, C19orf12, causes a distinct clinical subtype of neurodegeneration with brain iron accumulation. Am. J. Hum. Genet. 2011; 89: 543–550.

Haugarvoll K, Rademakers R, Kachergus JM, Nuytemans K, Ross OA, Gibson JM, et al. Lrrk2 R1441C parkinsonism is clinically similar to sporadic Parkinson disease. Neurology 2008; 70: 1456–1460.

Healy DG, Abou-Sleiman PM, Casas JP, Ahmadi KR, Lynch T, Gandhi S, et al. UCHL-1 is not a Parkinson's disease susceptibility gene. Ann. Neurol. 2006; 59: 627–633.

Healy DG, Abou-Sleiman PM, Lees AJ, Casas JP, Quinn N, Bhatia K, et al. Tau gene and Parkinson's disease: a case–control study and meta-analysis [Internet]. J. Neurol. Neurosurg. Psychiatry 2004; 75Available from: http://jnnp.bmj.com/content/75/7/962.full.pdf

Hernandez DG, Reed X, Singleton AB. Genetics in Parkinson disease: Mendelian versus non-Mendelian inheritance. J. Neurochem. 2016; 139: 59–74.

Jansen IE, Bras JM, Lesage S, Schulte C, Gibbs JR, Nalls MA, et al. CHCHD2 and Parkinson's. Lancet Neurol. 2015; 14: 678–679.

Kamm C, Fischer H, Garavaglia B, Kullmann S, Sharma M, Schrader C, et al. Susceptibility to DYT1 dystonia in european patients is modified by the D216h polymorphism. Neurology 2008; 70: 2261–2262.

Kasten M, Hartmann C, Hampf J, Schaake S, Westenberger A, Vollstedt EJ, et al. Genotype-Phenotype Relations for the Parkinson's Disease Genes Parkin, PINK1, DJ1: MDSGene Systematic Review. Mov. Disord. 2018; 33: 730–741.

Klopstock T, Elstner M, Lücking CB, Müller-Myhsok B, Gasser T, Botz E, et al. Mutations in the pantothenate kinase gene PANK2 are not associated with Parkinson disease. Neurosci. Lett. 2005;

379: 195–198.

Konno T, Ross OA, Teive HAG, Sławek J, Dickson DW, Wszolek ZK. DCTN1-related neurodegeneration: Perry syndrome and beyond. Park. Relat. Disord. 2017; 41: 14–24.

Köroĝlu Ç, Baysal L, Cetinkaya M, Karasoy H, Tolun A. DNAJC6 is responsible for juvenile parkinsonism with phenotypic variability. Park. Relat. Disord. 2013; 19: 320–324.

Krause P, Brüggemann N, Völzmann S, Horn A, Kupsch A, Schneider G-H, et al. Long-term effect on dystonia after pallidal deep brain stimulation (DBS) in three members of a family with a THAP1 mutation. J. Neurol. 2015; 262: 2739–2744.

Krebs CE, Karkheiran S, Powell JC, Cao M, Makarov V, Darvish H, et al. The sac1 domain of SYNJ1 identified mutated in a family with early-onset progressive parkinsonism with generalized seizures. Hum. Mutat. 2013; 34: 1200–1207.

Lautier C, Goldwurm S, Dürr A, Giovannone B, Tsiaras WG, Pezzoli G, et al. Mutations in the GIGYF2 (TNRC15) Gene at the PARK11 Locus in Familial Parkinson Disease. Am. J. Hum. Genet. 2008; 82: 822–833.

Lesage S, Patin E, Condroyer C, Leutenegger AL, Lohmann E, Giladi N, et al. Parkinson's disease-related LRRK2 G2019S mutation results from independent mutational events in humans. Hum. Mol. Genet. 2010; 19: 1998–2004.

Leuzzi V, Carducci C, Carducci C, Cardona F, Artiola C, Antonozzi I. Autosomal dominant GTP-CH deficiency presenting as a dopa-responsive myoclonus-dystonia syndrome. Neurology 2002; 59: 1241–1243.

Li YJ, Hauser MA, Scott WK, Martin ER, Booze MW, Qin XJ, et al. Apolipoprotein E controls the risk and age at onset of Parkinson disease. Neurology 2004; 62: 2005–2009.

Lill CM, Mashychev A, Hartmann C, Lohmann K, Marras C, Lang AE, et al. Launching the movement disorders society genetic mutation database (MDSGene). Mov. Disord. 2016; 31: 607–609.

Madadi F, Khaniani MS, Shandiz EE, Ayromlou H, Najmi S, Emamalizadeh B, et al. Genetic Analysis of the *ZNF512B* , *SLC41A1*, and *ALDH2* Polymorphisms in Parkinson's Disease in the Iranian Population. Genet. Test. Mol. Biomarkers 2016; 20: 629–632.

Malek N, Swallow DMA, Grosset KA, Lawton MA, Marrinan SL, Lehn AC, et al. Tracking Parkinson's: Study Design and Baseline Patient Data. J. Parkinsons. Dis. 2015; 5: 947–959.

Maraganore DM, Lesnick TG, Elbaz A, Chartier-Harlin MC, Gasser T, Krüger R, et al. UCHL1 Is a Parkinson's Disease Susceptibility Gene. Ann. Neurol. 2004; 55: 512–521.

Marder K, Maestre G, Cote L, Mejia H, Alfaro B, Halim A, et al. The apolipoprotein E4 allele in Parkinson's disease with and without dementia. Neurology 1994; 44: 1330–1331.

Martin ER, Scott WK, Nance MA, Watts RL, Hubble JP, Koller WC, et al. Association of single-nucleotide polymorphisms of the tau gene with late-onset Parkinson disease. JAMA 2001; 286: 2245–2250.

Marx FP, Holzmann C, Strauss KM, Li L, Eberhardt O, Gerhardt E, et al. Identification and

functional characterization of a novel R621C mutation in the synphilin-1 gene in Parkinson's disease. Hum. Mol. Genet. 2003; 12: 1223–1231.

Mencacci NE, Isaias IU, Reich MM, Ganos C, Plagnol V, Polke JM, et al. Parkinson's disease in GTP cyclohydrolase 1 mutation carriers. Brain 2014; 137: 2480–2492.

Myhre R, Klungland H, Farrer MJ, Aasly JO. Genetic association study of synphilin-1 in idiopathic Parkinson's disease. BMC Med. Genet. 2008; 9: 1–7.

Nichols N, Bras JM, Hernandez DG, Jansen IE, Lesage S, Lubbe S, et al. EIF4G1 mutations do not cause Parkinson's disease. Neurobiol. Aging 2015; 36: 2444–2444.e4.

Nichols WC, Kissell DK, Pankratz N, Pauciulo MW, Elsaesser VE, Clark KA, et al. Variation in GIGYF2 is not associated with Parkinson disease. Neurology 2009; 72: 1886–1892.

Nuytemans K, Rademakers R, Theuns J, Pals P, Engelborghs S, Pickut B, et al. Founder mutation p.R1441C in the leucine-rich repeat kinase 2 gene in Belgian Parkinson's disease patients. Eur. J. Hum. Genet. 2008; 16: 471–479.

Ohshima S, Tsuboi Y, Yamamoto A, Kawakami M, Farrer MJ, Kira J ichi, et al. Autonomic failures in Perry syndrome with DCTN1 mutation. Park. Relat. Disord. 2010; 16: 612–614.

Olgiati S, Quadri M, Fang M, Rood JPMA, Saute JA, Chien HF, et al. DNAJC6 Mutations Associated with Early-Onset Parkinson's Disease. Ann. Neurol. 2016; 79: 244–256.

Oliveira SA, Scott WK, Martin ER, Nance MA, Watts RL, Hubble JP, et al. Parkin mutations and susceptibility alleles in late-onset Parkinson's disease. Ann. Neurol. 2003; 53: 624–629.

Paisan-Ruiz C, Bhatia KP, Li A, Hernandez D, Davis M, Wood NW, et al. Characterization of PLA2G6 as a locus for dystonia-parkinsonism. Ann. Neurol. 2009; 65: 19–23.

Periquet M, Lücking C, Vaughan J, Bonifati V, Dürr a, De Michele G, et al. Origin of the mutations in the parkin gene in Europe: exon rearrangements are independent recurrent events, whereas point mutations may result from Founder effects. Am. J. Hum. Genet. 2001; 68: 617–626.

Purcell S, Neale B, Todd-Brown K, Thomas L, Ferreira MAR, Bender D, et al. PLINK: A tool set for whole-genome association and population-based linkage analyses. Am. J. Hum. Genet. 2007; 81: 559–575.

Quadri M, Fang M, Picillo M, Olgiati S, Breedveld GJ, Graafland J, et al. Mutation in the SYNJ1 gene associated with autosomal recessive, early-onset parkinsonism. Hum. Mutat. 2013; 34: 1208–1215.

Quadri M, Mandemakers W, Grochowska MM, Masius R, Geut H, Fabrizio E, et al. LRP10 genetic variants in familial Parkinson's disease and dementia with Lewy bodies: a genome-wide linkage and sequencing study. Lancet Neurol. 2018; 17: 597–608.

Risch NJ, Bressman SB, Senthil G, Ozelius LJ. Intragenic Cis and Trans Modification of Genetic Susceptibility in DYT1 Torsion Dystonia. Am. J. Hum. Genet. 2007; 80: 1188–1193.

Saunders-Pullman R, Fuchs T, San Luciano M, Raymond D, Brashear A, Ortega R, et al. Heterogeneity in primary dystonia: Lessons from THAP1, GNAL, and TOR1A in Amish-Mennonites. Mov. Disord. 2014; 29: 812–818.

Shojaee S, Sina F, Banihosseini SS, Kazemi MH, Kalhor R, Shahidi GA, et al. Genome-wide Linkage Analysis of a Parkinsonian-Pyramidal Syndrome Pedigree by 500 K SNP Arrays. Am. J. Hum. Genet. 2008; 82: 1375–1384.

Simón-Sánchez J, Singleton AB. Sequencing analysis of OMI/HTRA2 shows previously reported pathogenic mutations in neurologically normal controls. Hum. Mol. Genet. 2008; 17: 1988–1993.

Strauss KM, Martins LM, Plun-Favreau H, Marx FP, Kautzmann S, Berg D, et al. Loss of function mutations in the gene encoding Omi/HtrA2 in Parkinson's disease. Hum. Mol. Genet. 2005; 14: 2099–2111.

Tacik P, Fiesel FC, Fujioka S, Ross OA, Pretelt F, Castañeda Cardona C, et al. Three families with Perry syndrome from distinct parts of the world. Park. Relat. Disord. 2014; 20: 884–888.

Trinh J, Zeldenrust FMJ, Huang J, Kasten M, Schaake S, Petkovic S, et al. Genotype-phenotype relations for the Parkinson's disease genes SNCA, LRRK2, VPS35: MDSGene systematic review. Mov. Disord. 2018

Tucci A, Nalls MA, Houlden H, Revesz T, Singleton AB, Wood NW, et al. Genetic variability at the PARK16 locus. Eur. J. Hum. Genet. 2010; 18: 1356–1359.

Umemoto G, Tsuboi Y, Furuya H, Mishima T, Fujioka S, Fujii N, et al. Dysphagia in Perry Syndrome: Pharyngeal Pressure in Two Cases. Case Rep. Neurol. 2017; 9: 161–167.

Unal Gulsuner H, Gulsuner S, Mercan FN, Onat OE, Walsh T, Shahin H, et al. Mitochondrial serine protease HTRA2 p.G399S in a kindred with essential tremor and Parkinson disease. Proc. Natl. Acad. Sci. 2014; 111: 18285–18290.

Wang K, Li M, Hakonarson H. ANNOVAR : functional annotation of genetic variants from high-throughput sequencing data. 2010; 38: 1–7.

Wang L, Cheng L, Li NN, Yu WJ, Sun XY, Peng R. Genetic analysis of SLC41A1 in Chinese Parkinson's disease patients. Am. J. Med. Genet. Part B Neuropsychiatr. Genet. 2015; 168: 706–711.

Williams-Gray CH, Goris A, Saiki M, Foltynie T, Compston DAS, Sawcer SJ, et al. Apolipoprotein e genotype as a risk factor for susceptibility to and dementia in Parkinson's Disease. J. Neurol. 2009; 256: 493–498.

Wintermeyer P, Kruger R, Kuhn W, Muller T, Woitalla D, Berg D, et al. Mutation analysis and association studies of the UCHL1 gene in German Parkinson's disease patients. Neuroreport 2000; 11: 2079–2082.

Zabetian CP, Hutter CM, Yearout D, Lopez AN, Factor SA, Griffith A, et al. LRRK2 G2019S in families with Parkinson disease who originated from Europe and the Middle East: evidence of two distinct founding events beginning two millennia ago. Am. J. Hum. Genet. 2006a; 79: 752–8.

Zabetian CP, Morino H, Ujike H, Yamamoto M, Oda M, Maruyama H, et al. Identification and haplotype analysis of LRRK2 G2019S in Japanese patients with Parkinson disease. Neurology 2006b; 67: 697–699.

Zech M, Castrop F, Schormair B, Jochim A, Wieland T, Gross N, et al. DYT16 revisited: Exome

sequencing identifies PRKRA mutations in a European dystonia family. Mov. Disord. 2014; 29: 1504–1510.

Zittel S, Moll CKE, Brüggemann N, Tadic V, Hamel W, Kasten M, et al. Clinical neuroimaging and electrophysiological assessment of three DYT6 dystonia families. Mov. Disord. 2010; 25: 2405–2412.
